# Tryptophan and Non-Tryptophan Fluorescence of the Eye Lens Proteins Provides Diagnostics of Cataract at the Molecular Level

**DOI:** 10.1038/srep40375

**Published:** 2017-01-10

**Authors:** Anna Gakamsky, Rory R. Duncan, Nicola M. Howarth, Baljean Dhillon, Kim K. Buttenschön, Daniel J. Daly, Dmitry Gakamsky

**Affiliations:** 1 Edinburgh Instruments, 2 Bain Square, Livingston, EH54 7DQ, UK; 2 Institute of Biological Chemistry, Biophysics and Bioengineering, School of Engineering and Physical Sciences, Heriot-Watt University, Edinburgh, EH14 6, UK; 3 Princess Alexandra Eye Pavilion, Edinburgh and Centre for Clinical Brain Sciences, School of Clinical Sciences, University of Edinburgh, UK; 4 Lein Applied Diagnostics, Reading Enterprise Centre, Whiteknights Rd, Reading RG6 6BU, UK

## Abstract

The chemical nature of the non-tryptophan (non-Trp) fluorescence of porcine and human eye lens proteins was identified by Mass Spectrometry (MS) and Fluorescence Steady-State and Lifetime spectroscopy as post-translational modifications (PTM) of Trp and Arg amino acid residues. Fluorescence intensity profiles measured along the optical axis of human eye lenses with age-related nuclear cataract showed increasing concentration of fluorescent PTM towards the lens centre in accord with the increased optical density in the lens nucleolus. Significant differences between fluorescence lifetimes of “free” Trp derivatives hydroxytryptophan (OH-Trp), *N*-formylkynurenine (NFK), kynurenine (Kyn), hydroxykynurenine (OH-Kyn) and their residues were observed. Notably, the lifetime constants of these residues in a model peptide were considerably greater than those of their “free” counterparts. Fluorescence of Trp, its derivatives and argpyrimidine (ArgP) can be excited at the red edge of the Trp absorption band which allows normalisation of the emission spectra of these PTMs to the fluorescence intensity of Trp, to determine semi-quantitatively their concentration. We show that the cumulative fraction of OH-Trp, NFK and ArgP emission dominates the total fluorescence spectrum in both emulsified post-surgical human cataract protein samples, as well as in whole lenses and that this correlates strongly with cataract grade and age.

Cataract remains the leading cause of blindness in both developed and developing countries. According to the assessment of the World Health Organisation (WHO) cataract was responsible for 51% of the world blindness, which represented about 20 million people in 2010^1^. Currently the only treatment available for cataract is surgery. The aging population inevitably increases the number of people with cataract and hence the number of required operations.

Current cataract diagnosis is based on light scattering methods^2^ which can detect defects in the lens structure when they become comparable or greater than the optical wavelength, i.e. ~0.5 μm. However the formation of micron-size defects is preceded, and likely to be caused, by the formation of multiple PTMs in the eye lens crystallin proteins^3^,^4^,^5^,^6^. Hence, monitoring the formation of PTMs in crystallins has the potential to elevate cataract diagnostics to the molecular level and develop a more sensitive method applicable in the latent phase of the disorder, as well as providing a mechanistic insight into cataractogenesis. It might be also used for *in vitro* or *ex vivo* drug screening applications and for monitoring changes in the lens structure in the course of administration of cataract cure medications.

The eye lens grows layer by layer throughout the lifespan of the organism. Matured eye lens fibre cells do not contain organelles apart from the proteasome^7^. The absence of organelles gives the eye lens optical homogeneity and minimises Rayleigh scattering. This means that protein synthesis is impossible and therefore the proteins synthesised in new cells remain in mature cells for their entire lifespan. The absence of protein turnover brings about the accumulation of a large number of PTMs in crystallins, resulting in protein misfolding and aggregation^8^, which in turn increases light scattering. In addition, some PTMs absorb light in the visible spectral range thereby reducing lens transparency and giving rise to coloration. These two effects are mainly responsible for cataract.

Oxidation of the Trp side chain, indole, affects Trp fluorescence directly by decreasing its intensity and forming a range of aromatic products with absorption and emission in the visible spectral range. Modifications of nearby side chains can affect Trp fluorescence indirectly by changing the protein structure and altering micro-polarity and quenching conditions. Such modifications can change the position of the Trp emission spectrum and fluorescence lifetime. Hence, the formation PTMs in crystallins endows the eye lens a unique fluorescence “signature” that may be measurable.

Molecular mechanisms of tryptophan oxidation have been studied in model peptides and proteins^9^,^10^,^11^ and a progressive increase in the rate of Trp degradation from proteins to peptides and further to free Trp was observed. A model of protein Trp depletion via oxidation, resulting in protein pigmentation has been suggested^12^. The largest fraction of non-Trp fluorescence is found to be associated with the insoluble protein fraction of the eye lens proteins^13^,^14^. The increase in the lens optical density and, in part, in non-Trp fluorescence, has been attributed to the formation of 3-hydroxykynurenine glucoside^15^, 4-(2-amino-3-hydroxyphenyl)-4-oxobutanoic acid^16^, and glutathione-3-hydroxykynurenine glycoside^17^ which can also cross-link the lens proteins causing the formation of high molecular mass aggregates.

Non-Trp fluorescence or autofluorescence has been extensively used to quantify cataractous changes in the eye lens^12^,^14^,^18^,^19^,^20^,^21^,^22^,^23^,^24^,^25^. It has been shown that an increase in non-Trp fluorescence correlates with an increase in the concentration of both the 360 nm and 435 nm fluorophores (emission maxima at 420–440 nm and 500–520 nm respectively) and the second species is likely to be a be a product of the first one^18^. Evidence has also been presented that lens autofluorescence increases with age and the development of diabetes^26^,^27^. However, some results reported similarity in the fluorescence properties of lenses with different grade of cataracts, finding that the spectral properties of autofluorescence remains unaltered by age and diabetes^23^ or that the intensity of the blue autofluorescence does not correlate with dose of UV radiation^24^.

This apparent controversy in the literature may result from the complexity of fluorescence measurements in the eye lens. Fluorescence intensity is a function of various experimental parameters, such as geometry of the excitation and emission channels, intensity of excitation light, optical density at the excitation wavelength etc. In addition, fluorometric measurements conducted in an extracted eye lens or on a whole eye are complicated by the presence of stray light, reflections on the cornea and/or other interfaces, and the inner filter effect caused by high Trp concentration and elevated concentration of non-Trp species^28^. To become quantitative or semi-quantitative such a method requires a reference signal which is independent of the concentration of PTMs and varies by experimental factors in the same proportion as the emission of PTMs. To this end Lerman and Borkman^18^ used Trp fluorescence excited at 290 nm to normalise intensities of the 360 nm and 435 nm fluorophores. The use of 290 nm excitation, however, is not fully ideal because of the high optical density of the eye lens which greatly attenuates 290 nm light and permits excitation of the lens surface only. The normalisation of non-Trp fluorescence from inner regions on the intensity of surface emission of Trp cannot correct for the inner filter effect and hence the concentration of PTM in inner regions may be underestimated.

In this work we show that the limitation caused by high Trp concentration can be circumvented by exciting the lens on the red edge of the Trp absorption spectrum where young or normal lenses are relatively transparent. Using MS we identified the chemical nature of non-Trp emission in several cataract models as the formation of fluorescent adducts of Trp and Arg and confirmed their presence in eye lens proteins by fluorescence lifetime measurements. We also show that the emission spectrum of a cataractous lens can be decomposed over the emission spectra of fluorescent PTMs. The calculated fractions of individual PTMs being normalised on fluorescence intensity of Trp yield a semi-quantitative diagnostics of cataract.

## Results

### A young porcine eye lens is transparent at the 317 nm wavelength

**Figure 1:**
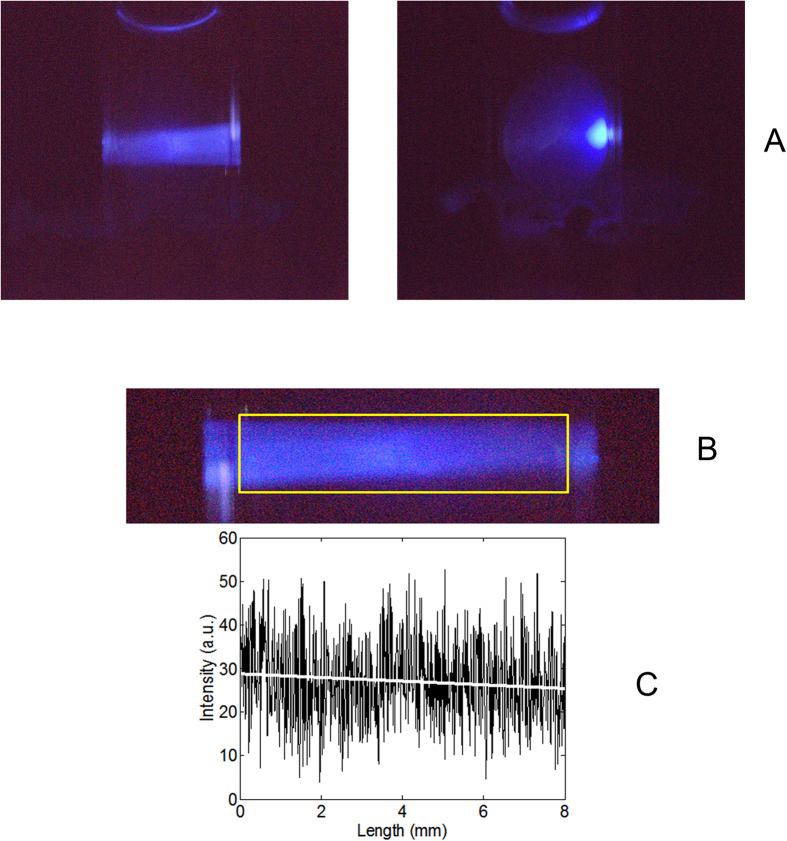
(**A**) Excitation of eye lens fluorescence in a normal (left) and UV-irradiated porcine lens by a 317 nm-LED. (**B**) A zoomed trace of the 317 nm-beam in the normal lens. (**C**) Analysis of the change in the fluorescence intensity of the excitation beam.

Due to the high concentration of crystallins in the eye lens (200–400 mg/ml) optical density in the 270–300 nm range is very high and hence just fluorescence of the lens surface proteins can be excited in this spectral range. However, the lens optical density quickly drops at the red edge of the Trp absorption spectrum and the light beam of a 317 nm-LED penetrates with only ~25% attenuation (Fig. 1A and B), as evaluated by densitometry (Fig. 1C). This attenuation is equal to an optical density (OD) of 0.13.

In contrast, the excitation beam is fully absorbed by the damaged volume formed in the lens upon exposure to intense UV irradiation (Fig.1A, right panel) and fluorescence intensity emitted by the damaged volume greatly surpasses that of the control lens (A, left panel). These data show that the 317 nm wavelength can be used for 3D fluorescence mapping of the eye lens.

To gain further insight into the nature of non-Trp fluorescence several models have been used: cataracts induced by UV irradiation in a normal porcine lens, UV-irradiated solubilised porcine lens protein samples, emulsified human donor lens samples received after cataract surgery and intact human donor eye lenses.

### Fluorescence emission map of donor eye lenses and soluble crystallins

**Figure 2:**
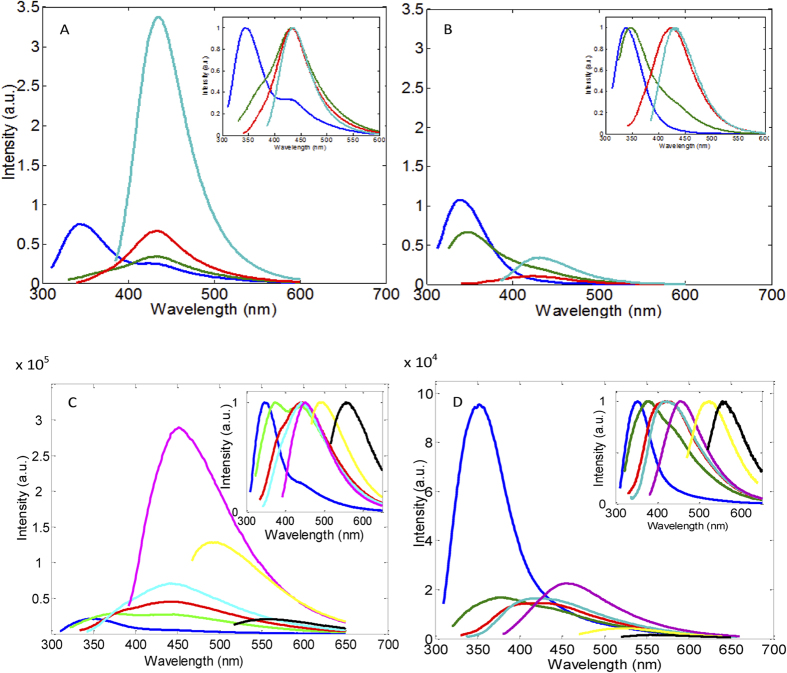
Emission spectra of a UV-irradiated (**A**) and normal porcine eye lens (**B**) measured at 305 nm (blue), 317 nm (green), 325 nm (red) 370 nm (cyan) excitation wavelengths. The respective normalised spectra of the normal and UV-irradiated lenses are shown in the Insets. Emission spectrum map of solubilised porcine lens after UV irradiation (**C**) and the insoluble fraction of an emulsified donor sample (NC++) (**D**) upon 300 nm (blue), 317 nm (green), 325 nm (red) 370 nm (cyan), 400 nm (magenta), 450 nm (yellow) and 500 nm (black) excitation wavelengths. Normalised spectra of the UV-irradiated and the emulsified lens (Insets).

Fluorescence emission maps measured from an exemplar damaged volume and an exemplar control sample are shown in Fig. 2A and B, respectively. One can see that light of wavelength 305 nm excited Trp emission in the control lens (blue), whereas light of wavelength 317 nm also excited non-Trp emission which appeared as a shoulder with maximum at ~435 nm on the red slope of the Trp emission spectrum (green). A similar non-Trp fluorescence band with larger amplitude was observed in the emission of the damaged lens (A). We observed only one rather broad band with a maximum at 435 nm (red) upon 325 nm excitation. A further increase in the excitation wavelength to 370 nm was found to decrease the spectral width in both samples at the expense of the blue slope; however the emission maximum remained unchanged (A and B, inset).

On comparing the emission maps recorded for the normal and irradiated lenses under identical experimental conditions, it was concluded that the intense emission generated by the damaged volume upon 317 nm LED excitation (Fig. 1) was caused by the 435 nm fluorophore.

UV-irradiated solubilised proteins of a porcine lens showed a significantly broader emission map covering the whole visible spectral range (C). A similar spectral coverage, but with a substantially reduced emission, was observed for an emulsified cataractous human sample classified with a second grade nuclear cataract (NC++) (D, note 10-fold scaled down Y-axis). The above results suggest that these samples share similar fluorophores but that concentration of them in the cataractous human lens samples was lower than in the porcine lens model.

### Identification of the chemical nature of non-Trp emission

Size exclusion chromatography was employed to analyse the size-distribution of crystallins in control and UV-irradiated solubilised porcine lens samples (Supplemental Fig. S1). Given that equal amounts of the protein obtained from each sample were examined, it was surprising to note a 60% increase in the area under the chromatogram for the irradiated sample relative to the control sample. The greatest change after irradiation was observed for the first peak, which corresponds to the heaviest (~400 kD) protein fraction. The observed ~5-fold increase in peak amplitude can be rationalised, in part, by the binding of smaller molecular mass proteins (β and γ-crystallins) to the heavy oligomeric complexes of α-crystallin as evident from the decrease in the amplitudes of the 2^nd^, 3^rd^ and 4^th^ peaks. This effect is expected as α-crystallins are known to bind denatured β- and γ- crystallins owing to their chaperone-like function. However, the disproportionate change in the 1^st^ peak’s amplitude compared to the amplitude changes of the other peaks and the 60% increase in the total area under the chromatogram suggest that additional chromophores were formed in the irradiated sample.

The above results led us to analyse the proteins in the 1^st^ peak by MS. Both samples showed multiple modifications, however modification rates were significantly higher in the irradiated sample (Supplemental Fig. S2). The MS data revealed relatively frequent modifications to the side chains of Met, followed by His, Cys, Trp, and less commonly Ala and Arg. The conversion of Gln to pyroglutamate (pyroGlu) and acetylation of Ser were also observed. Furthermore, besides the heavy α-crystallin oligomers, the lower molecular mass β/γ-crystallins were found in these fractions too, confirming the size exclusion conclusions regarding protein composition of the first peak. This finding demonstrates that α-crystallins form stable complexes with β/γ-crystallins and lends support to reports detailing the chaperone-like function of α-crystallins^29^,^30^.

Based on the modification rates we conclude that OH-Trp and NFK are primary products of Trp whereas Kyn is a secondary one.

PTMs in the soluble protein fractions of an emulsified human lens, classified with NC++ cataract, were subsequently investigated by MS. Modification rates of PTMs are summarised in Table 1. These were found to be significantly reduced compared to the UV-irradiated porcine model suggesting that oxidation proceeded more efficiently in the liquid protein sample upon exposure to UV-irritation than in the intact eye lens controlled by natural metabolism. In addition to the modifications which had been noted previously in the UV-irradiated model sample, two further PMTs were identified; those of OH-Kyn and ArgP. The latter was not observed in the UV-irradiated sample apparently because its formation requires interaction with methylglyoxal^31^ which was not present in this sample. The lack of detected OH-Kyn in the irradiated sample suggests that its formation requires specific conditions which were not provided in the irradiation experiment.

α-Crystallin B contains two Trp residues (W9 and W60). Our MS data showed a significantly lower modification rate for W9 than W60 in both the porcine and human samples. This is apparently a result of the different accessibility of the side chains of these Trp side chains to reactive oxygen species (ROS). This finding supports the predictions of a pseudoatomic model of a 24-meric αB-crystallin assembly obtained by a triple hybrid approach combining data from cryo-electron microscopy, NMR spectroscopy, and structural modelling^32^ which suggests that W9 is more protected from aqueous environment than W60. The data also show a remarkably high rate of Arg modification in α-crystallins A and B, 6.1% and 10.7%, respectively, with nearly 100% modification of R120 being observed in α-crystallin B, known to play an important role in the chaperon-like function of these proteins^33^.

Summarising the aggregated MS data, we note that in total, we identify five abundant fluorescent PTMs: OH-Trp, NFK, Kyn, OH-Kyn and ArgP modification rates of which vary from a fraction of per cent to ~11% which in turn corresponds to the millimolar concentration range.

### Fluorescence properties of the Trp derivatives and ArgP

**Figure 3:**
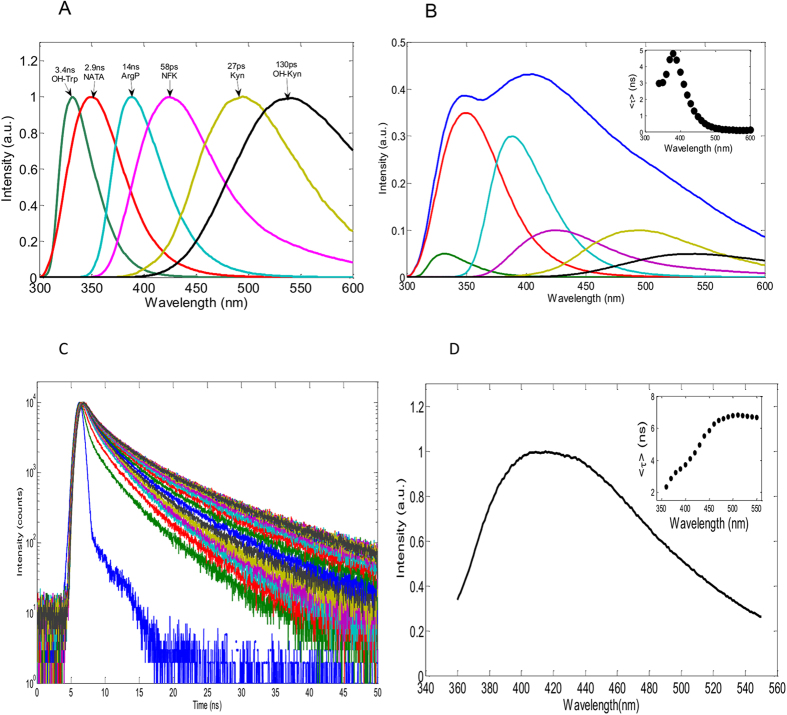
Fluorescence spectral and lifetime parameters of Trp, its derivatives and ArpP (**A**). A simulated emission spectrum generated from emission spectra of Trp and the OH-Trp, ArgP, NFK, Kyn and OH-Kyn in the 5:37:11:32:11:5 proportion (**B**) and spectral dependence of average fluorescence lifetime (inset). A typical example of fluorescence lifetime responses of the insoluble fraction of an emulsified donor eye lens (NC++) measured across the emission spectrum in the 350–550 nm range upon 317 nm excitation (**C**). Steady-state emission spectrum of this sample upon 317 nm excitation (**D**) and a spectral dependence of averaged lifetime calculated from the data shown in the left panel (inset).

Amino acid counterparts of the identified PTMs were used to measure their emission spectra and lifetime constants (Fig. 3A). The spectral position of NFK emission suggests that it is likely to be responsible for the 435 nm band observed in the emission spectrum of porcine lenses (Fig. 2A). As the OH-Trp emission spectrum is blue-shifted from the emission spectrum of Trp it could not be seen upon 317 nm excitation. On the other hand, NFK, Kyn, OH-Kyn and ArgP are likely to contribute to the non-Trp emission in the >400 nm range.

The above findings imply that the eye lens fluorescence is caused by emission of at least five fluorophores, including Trp, with their spectra predominantly overlapping. To better understand their relative contributions, we employed fluorescence lifetime measurements. The lifetime constants measured for these molecules are listed in Fig. 3A and the raw data are shown in Supplemental Fig. S3. One can see that NATA, OH-Trp and ArgP emit in the nanosecond range whereas the longer wavelength fluorophores NFK, Kyn and OH-Kyn emit in the sub-nanosecond range. Thus, given these results, it was reasoned that if the proposed hypothesis about the nature of eye lens emission in the visible spectral range was correct, then the emission should have sub-nanosecond lifetimes in the >450 nm range. This notion is illustrated by simulated data shown in Fig. 3B. In this simulation we calculated a steady-state fluorescence spectrum (blue) as a weighted sum of the individual spectra of each of these components. Relative weights of the components were chosen such to get a spectrum, which resembles the spectral shape observed for the human sample (Fig. 2D, green). Then average fluorescence lifetime constants along the spectral range were calculated as weighted sums of the lifetime constants using the spectral intensities of the individual components as the weighting factors (inset). As expected, the simulated lifetime data have nanosecond values in the 320–450 nm range and picosecond values in the >450 nm range.

This simulation contrasts drastically with the observed lifetime data (Fig. 3C) measured in the emulsified cataractous samples. Evaluation of these data (Supplemental Fig. S4) requires at least four lifetime components to get a satisfactory “global” fit. Average lifetimes calculated from these data plotted as a function of wavelength (Fig. 3D, inset) show a substantial (~3.5-fold) increase from ~2 ns on the blue slope to ~7 ns on the red slope of the emission spectrum. In other words we observe two orders of magnitude discrepancy between the average fluorescence lifetime of ”free” Kyn (60 ps) and ~7 ns lifetime of the protein sample in the green spectral range where emission of Kyn dominates.

To rationalise this discrepancy we hypothesised that the fluorescence properties of the “free” Trp derivatives differ from those of the corresponding amino acid residues incorporated into peptides or proteins. To test this hypothesis we investigated the fluorescence properties of Trp and its derivatives in a model peptide.

### Fluorescence properties of Trp and its photoproducts in AAWAA peptide

**Figure 4:**
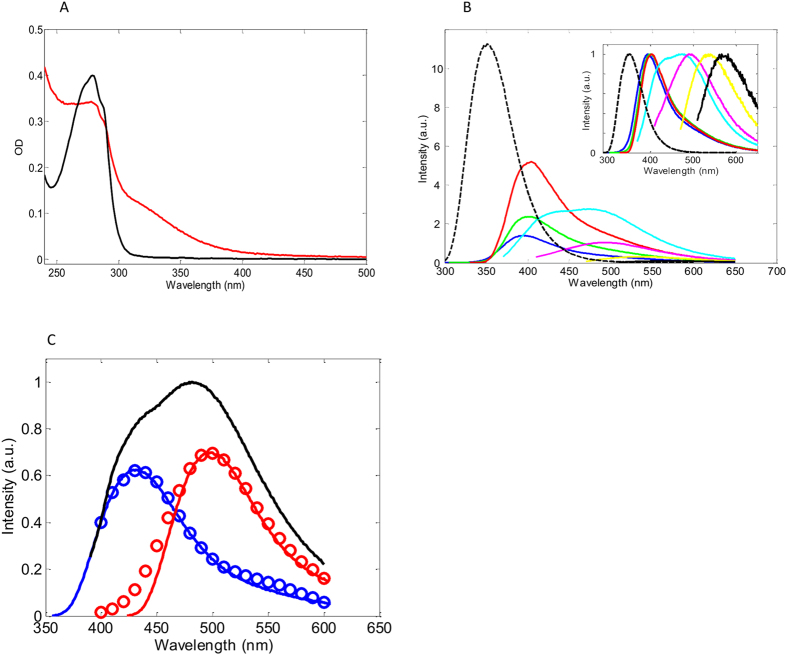
Absorption spectra of AAWAA before (black) and after (red) UV irradiation (**A**). Emission spectra of AAWAA before UV irradiation upon 280 nm excitation (dash black), and after UV irradiation upon 280 nm (blue), 300 nm (green) 320 nm (red), 350 nm (cyan), 400 nm (magenta), 450 nm (yellow) and 500 nm (black) excitation (**B**). Normalised emission spectra (inset). Decomposition of the emission spectrum of Fr5 measured upon 372 nm excitation (black) using spectra associated with τ_1_,τ_2_ and τ_3_ (blue circles) and τ_4_ (red circles) lifetime components. The parameters were calculated by evaluating fluorescence time-responses of Fr5 in the 400–600 nm range by a 4-exponential model with “linked” lifetime parameters. (**C**) Blue-shifted by 5 nm fluorescence spectra of NFK (blue) and fluorescence spectrum of Kyn (red) in PBS.

We used a model 5aa peptide containing one Trp to photo-derivatise its Trp side chain using intensive UV light. Figure 4A shows absorption spectra of a control and irradiated samples. One can see that the UV-irradiation affected the shape of the 280 nm band and formed an additional absorption band on the red side of the Trp absorption spectrum. It is interesting that the emission map of the irradiated peptide (Fig. 4B) was similar to those observed for the irradiated porcine lens proteins and the emulsified human donor samples (Fig. 2C and D). The similarity of these emission maps and the formation of the absorption spectrum in the visible range suggest that tryptophan photo-derivatives could be, at least partially, responsible for the non-Trp fluorescence and increasing absorption in the eye lens.

The irradiated peptide was subjected to reversed-phase HPLC fractionation as shown by a chromatogram in Supplemental Fig. S5. The fractions were analysed by MS (Supplemental Fig. S6) and the fractions containing Trp photo-derivatives were characterised spectroscopically.

The fluorescence time-response of the starting peptide was found to be bi-exponential with 1.1ns (34.4%) and 2.0 ns (66.6%) lifetime constants (Supplemental Fig. S3b), whereas the emission response of NATA was mono-exponential with 2.9 ns lifetime constant (Supplemental Fig. S3a).

We found that the emission spectrum of the fraction containing AA-OH-Trp-AA (Fr. 2) was 45 nm red-shifted compared with the emission spectrum of “free” OH-Trp and its average fluorescence lifetime of 4.5 ns was ~30% longer than that of OH-Trp (3.4 ns) (Supplemental Fig. S3c and d).

The fraction containing AA-OH-Kyn-AA (Fr. 8) showed a similar emission spectrum to “free” OH-Kyn (Fig. 3A and Supplemental Fig. S3i) however its average lifetime of 4.4 ns was 14-fold longer (Supplemental Fig. S3j, left) than the average lifetime of its free counterpart (Supplemental Fig. S3j, right).

Unfortunately, this chromatography could not resolve AAA-NFK-AA and AA-Kyn-AA and both these products were co-eluted in Fr. 5. Therefore, unsurprisingly, the normalised steady-state emission spectrum of this fraction (Fig. 4C, black) was a composite of the two unresolved emission bands. A spectral decomposition is described in Supplemental Section A. Spectra associated with the first three lifetime components (blue circles) and the fourth component (red circles) were attributed to emission of Kyn and NFK.

Finally, we measured fluorescence spectra and lifetimes of ArgP and pentosidine (Supplemental Fig. S3k,l). Both these molecules can be excited efficiently with 317 nm light and have similar emission spectra with maxima at 389 nm and 375 nm respectively. The fluorescence lifetime responses of these molecules were mono-exponential with 14.1 ns and 4.1 ns lifetime constants respectively.

The fluorescence lifetime constants of Trp derivatives measured in this work are summarised in Supplemental Table S1.

### Spectral analysis of emulsified eye lens proteins

**Figure 5:**
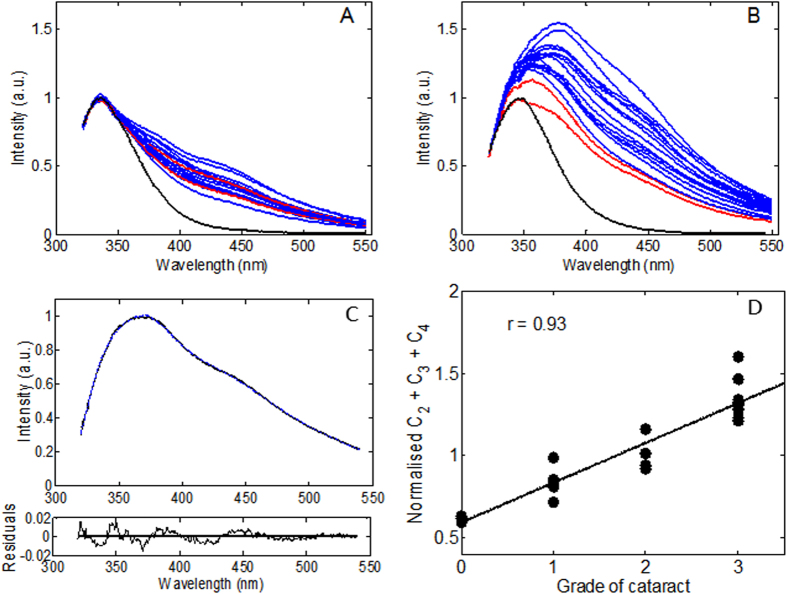
Fluorescence spectra of the soluble (**A**) and insoluble (**B**) fractions of emulsified donor eye lens proteins measures upon 310 nm excitation. The spectra were normalised on the emission spectrum of Trp (black) measured in samples of porcine eye lens proteins solubilised either in PBS (left panel, black) or in PBS/7 M urea buffer (right panel, black) upon 310 nm excitation. (**C**) Decomposition of the emission spectrum (excitation 310 nm) of the insoluble fraction of an emulsified cataractous sample (NC++) (black) over fluorescence spectra of NATA (1), AA-OH-Trp-AA (2), ArgP (3), AA-Kyn-AA (4), AA-Kyn-AA (5) and AA-OH-Kyn-AA (6) (blue) and corresponding fit-function parameters: C_1_ = 0.84, C_2_ = 0.21, C_3_ = 0.20, C_4_ = 0.46, C_5_ = 0.05, C_6_ = 0.08. Residuals function (bottom panel). The spectral decomposition was carried out using equation (4). Correlation of cataract grade with the normalised cumulative fraction (C_2_ + C_3_ + C_4_)/C_1_ for 21 emulsified eye lens samples (**D**).

Emulsified in the course of cataract surgery eye lens proteins were separated into soluble and insoluble fractions by centrifugation. The soluble fractions were concentrated by a centrifugal filter and the pellets were re-suspended in 8 M urea for further spectroscopic analysis. The spectra of the soluble and insoluble fractions were normalised on the Trp emission intensity as shown in Fig. 5.

The intensity of non-Trp fluorescence in the insoluble fractions (Fig. 5B) was found to be significantly greater than that of the soluble ones (Fig. 5A). Importantly, the emission spectra of the control soluble fractions were found to be statistically indistinguishable from those of the cataractous samples, whereas in the insoluble fractions the spectral controls consistently showed lower amplitudes.

Fluorescence spectra of the insoluble fractions of 21 human donor samples, with different grades of cataracts, were decomposed over the six elementary spectral components identified earlier. Figure 5C shows a typical example of spectral decomposition of a cataractous sample (NC++). The fractional coefficients, *C*_*i*_, (equation (4)) calculated in the course of spectral decomposition give relative concentrations of the respective spectral components (*C*_*1*_ − Trp, *C*_*2*_ − OH-Trp, *C*_*3*_ − ArgP, *C*_*4*_ − NFK, *C*_*5*_ − Kyn, and *C*_*6*_ − OH-Kyn). Although these coefficients are relative they can be normalised on the fractional coefficient of Trp, as its concentration was almost constant in different lenses in accord with the 1–2% modification rate obtained in our MS experiments. Such normalisation therefore allows the fractional coefficients to be compared across different samples. We found that the cumulative emission of OH-Trp, ArgP and NFK dominated the total fluorescence spectrum in agreement with our MS data. Figure 5D shows that the normalised cumulative coefficient (*C*_*2*_ + *C*_*3*_ + *C*_*4*_)/*C*_*1*_ representing the dominant fluorescent fraction correlates well with cataract grade with a Pearson’s coefficient (r) of 0.93.

### Spectral analysis of fluorescence emission of whole donor lenses

Having established the molecular identities and proportional contributions of all the key fluorescent species in the lens that develop during cataractogenesis, decomposition of the total fluorescence spectrum recorded for an intact lens was next examined in order to ascertain if such an approach could potentially be used as a diagnostic for human cataract.

**Figure 6:**
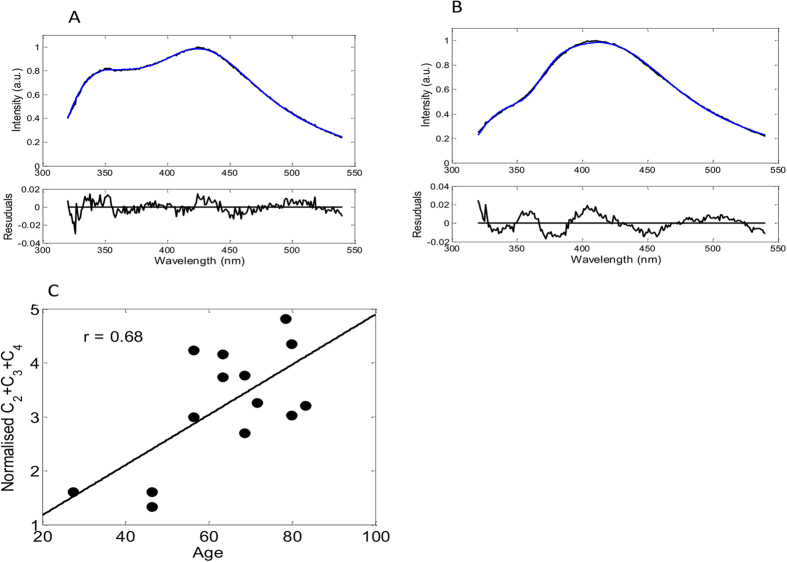
Decomposition of emission spectra (excitation 317 nm) of a 30 (**A**) and 80 years old (**B**) donor lenses over fluorescence spectra of NATA (1), AA-OH-Trp-AA (2), ArgP (3), AA-Kyn-AA (4), AA-Kyn-AA (5n) and AA-OH-Kyn-AA (6). Fit-functions (blue) with C_1_ = 0.74, C_2_ = 0.14 C_3_ = 0.14, C_4_ = 0.84, C_5_ = 0.03 C_6_ = 0.12 (panel A) and C_1_ = 0.42, C_2_ = 0.12, C_3_ = 0.39, C_4_ = 0.72, C_5_ = 0.00, C_6_ = 0.09 (panel B). Residuals functions (**A**,**B** bottom panels). The spectral decompositions were carried out using equation. (4). Correlation of age with normalised cumulative fraction (C_2_ + C_3_ + C_4_)/C_1_ for 14 donor lenses with age related nuclear cataracts (**C**).

Emission spectra of 17 human lenses from donors aged 30–83 were measured. We found that these emission spectra can also be decomposed using the set of spectral functions described earlier. However, in order to obtain a satisfactory fit the positions of OH-Trp emission spectra had to be blue-shifted by 4 nm. This adjustment accounts for the difference in polarity of this side chain in PBS and folded crystallins. Figure 6A and B show examples of spectral decomposition of relatively young (30 yo) and old (80 yo) donor lens samples. It can be seen that the relative contribution of non-Trp fluorescence in the emission of the old lens is significantly greater than that in the emission of the young one. As before, the cumulative emission of OH-Trp, ArgP and NFK was found to be dominant in all samples. Figure 6C shows a correlation of the normalised cumulative coefficient (*C*_*2*_ + *C*_*3*_ + *C*_*4*_)/*C*_*1*_ with age. Pearson’s correlation coefficient for these data was calculated as r = 0.68. Although these data show statistically significant correlation, the smaller value of the correlation coefficient suggests that age is not a unique factor affecting lens fluorescence. For example, we found that the non-Trp emission intensity from the left and right eyes of the same 83 yo donor differed by ~25%.

### Distribution of PTM in the eye lens

**Figure 7:**
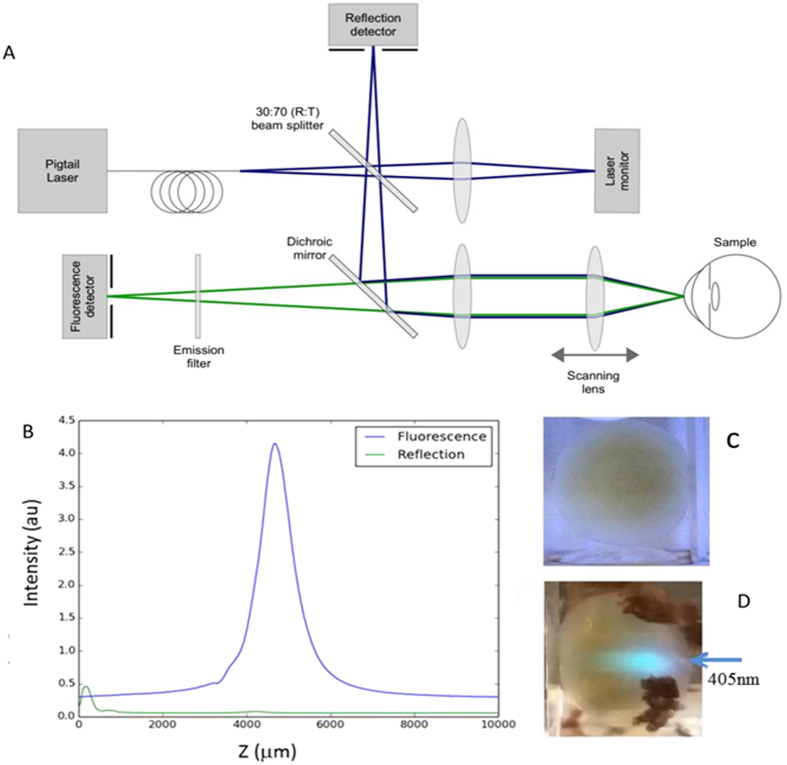
(**A**) Confocal fluorescence microscope F410. The excitation beam of a 405 nm single mode fibre-coupled laser diode (LP405-SF10, Thorlabs, NJ. USA) is first split into the 30:70 (R:T) proportion by the beam splitter before being reflected by a dichroic mirror (cut-on 425 nm). The light is then being collimated by an achromatic doublet lens (f = 200 mm) and focused on the sample by an achromatic doublet and meniscus lens combination (f = 47.11 mm) mounted on a motorised translation stage (SMAC Europe, UK). Fluorescence and scattered signals collected from the sample by the scanning and collimating lenses are separated by the dichroic mirror. The scattered and fluorescence signals are focused onto a photo diode (PDA36A-EC, Thorlabs) through a 150 μm pinhole a photomultiplier tube module (H9306-04, Hamamatsu) through an emission filter (cut-on 450 nm) and a 50 μm pinhole respectively. Laser intensity is measured using the 70% portion of the excitation beam by a photo diode (PDA100A-EC, Thorlabs). Digitized by an analogue-to-digital converter (ADC) (USB-1208FS, Measurement Computing, MA, USA) intensities of the fluorescence and scattered light signals were normalised on the excitation intensity by the instrument software and plotted as functions of scanning lens position (Z). (**B**) Fluorescence intensity distribution profile along the optical axis carried out in a in a donor eye lens with age related nuclear cataract correlate with increased optical density in the nucleus. (**C**) Density of the yellow coloration increases towards the nucleus. (**D**) The experimental geometry of the fluorescence intensity distribution measurements.

Having found that fluorescent PTMs are predominantly associated with the insoluble fraction of crystallins, its distribution in the lens was investigated. To this end we employed a confocal fluorescence microscope (Fig. 7A). The 405 nm-laser used in this microscope cannot excite the emission of Trp and OH-Trp and ArgP and the excitation of NFK by this wavelength is also very unfavourable. Thus, out of the six fluorescent PTMs only Kyn and OH-Kyn were predominantly excited. The instrument measured the total emission of these molecules through a long path >425 nm optical filter. A typical bell-shaped fluorescence intensity profile along the optical axis of the lens is shown in Fig. 7B. Figure 7C shows that the density of the yellow coloration increased towards the nucleus. Figure 7D shows the measurement geometry. The bell-shaped intensity profile observed for this lens was typical for all 14 donor lenses with age-related nuclear cataract. These data suggest that concentration of fluorescent PTMs and hence concentration of the insoluble fraction proteins is highest in the lens nucleus for this type of cataract.

## Discussion

Here, we present a comprehensive characterisation of the fluorescence properties of PTMs accumulated in the eye lens proteins. We resolve discrepancies in the literature, demonstrating that these PTMs predominantly occur in polypeptide chains as opposed to in ‘free’ amino acids. We also present a novel analytical tool for the decomposition of complex fluorescence spectra acquired non-invasively from human lenses and conform it by fluorescence lifetime spectroscopy.

The eye lens contains very high concentrations (200 – 400 mg/ml) of protein comprising 30% α-, 55% β- and 15% γ- crystallin^34^. On average, one molecule of crystallin contains 4 Trp residues resulting in ~60 mM of Trp at the 300 mg/ml concentration of protein. For this concentration, using the extinction coefficient of Trp ε(280 nm) = 5,600 M^−1^ cm^−1^ we get OD = 336 at the maximum of the Trp absorption spectrum, which attenuates light by 99% in a 30 μM surface layer. However, the red slope of Trp absorption spectrum is very steep and the extinction coefficient decreases 14-fold at 305 nm, 100-fold at 310 nm and by about 1000-fold at 317 nm. This gives an OD (317 nm) of ~0.34. Our data on the transmission of 317 nm light yielded an OD (317 nm) of ~0.13 in 5 mm lens, i.e. 0.26 in 1 cm, which agrees with the above estimate. Hence the 317 nm wavelengths can excite fluorescence in the lens interior and therefore can be used for 3D fluorescence mapping of the lens.

Trp is a polarity sensitive fluorescence probe which implies that positions of its absorption and emission spectra are functions of polarity^35^. In a multi-tryptophan protein each Trp side chain is situated in a unique microenvironment (“pocket”) characterised by a particular polarity. Some pockets are relatively hydrophobic (low polarity) whereas others are hydrophilic (high polarity). Usually the model of this hydrophilic pocket corresponds to a surface-exposed Trp side chain surrounded by water molecules. Due to the spectral selectivity on the red edge of the Trp absorption spectrum, one can excite a fraction of Trp residues situated in hydrophilic pockets with a red-shifted absorption spectrum^35^. The emission spectrum of this fraction has a maximum at 350 nm (Fig. 2B). Excitation with 295 nm light excites a Trp fraction situated predominantly in hydrophobic pockets with an emission maximum at 333 nm. Excitation at 305 nm gives an emission maximum at 338 nm.

Together with the excitation of the “polar fraction” of Trp, red edge excitation also excites emission of non-Trp fluorescence appearing as a small shoulder on the red emission slope with a maximum at ~435 nm. The spectral position of this unresolved band suggests that it results from the emission of OH-Trp and NFK residues.

The emission spectra of “free” Trp derivatives (NFK, Kyn and OH-Kyn) suggest that they are responsible for non-Trp fluorescence; however the actual spectral attribution was not straightforward due to the drastic difference between the fluorescence lifetime constants of the “free” fluorophores and their corresponding residue counterparts as illustrated in Fig. 3B and D inset. Our fluorescence lifetime data shed light on this discrepancy.

The fluorescence response of NATA is described by a mono-exponential function (Supplemental Fig. S3a); however, notably, emission of Trp residue in the mono-Trp peptide AAWAA is best described by a bi-exponential function (Supplemental Fig. S3b). The presence of two lifetime components in the fluorescence decay of this peptide is likely to be a result of emission of two different conformations of the Trp side chain^36^. Furthermore, we observed a single-exponential fluorescence decay of NATA and a double-exponential decay for the mono-Trp peptide when both are fully solvated by water molecules, i.e. in a microenvironment with similar polarity. This suggests that the attribution of the two fluorescence lifetime components of α-crystallin B time-response to the emission of W9 and W60, known to have different accessibility to aqueous environment^21^ seems rather unlikely.

The 1.7 ns average lifetime of Trp residue and 4.5 ns of OH-Trp reside in the peptide well agree with the average lifetime of 2.2 ns at 360 nm in the emission time-response of the emulsified cataractous sample, where emission spectra of these fluorophores overlap.

UV light-stimulated oxidative processes increased concentration of the above Trp photo-products in the lens (Fig. 2A). Likewise, UV light caused the formation of these products in solubilised protein samples with a wider range of fluorophores, evident from the emission spectrum map (Fig. 2C). Similar emission maps were also observed in the UV-irradiated peptide (Fig. 4B) and in emulsified cataractous human lenses (Fig. 2D). The similarity of emission maps suggests a generic nature of non-Trp fluorescence originating from emission of Trp and Arg photo-degradation products.

The fluorescent PTMs identified by MS do not cover the list of all possible fluorescent PTMs in eye lens proteins. Thus, Kessel *et al*.^23^ discussed the role of pentosidine, a pentose-formed cross-link between Lys and Arg, in non-Trp emission of the eye lens. Our MS analysis could not identify this PTM because of a lack of current analytical tools to access this PTM, and therefore we do not know the abundance of this PTM in crystallins. However, its concentration was found to be significantly lower than that of ArgP in tissue proteins^37^. One can suggest this is due to the third order of the chemical reaction involved in the formation of pentosidine, the rate of which is proportional to concentrations of pentose and surface exposed Lys and Arg. Considering the formation of this modification as a cross-link between two proteins, we suggest that the yield of this reaction is controlled by rotational diffusion of crystallins which is a relatively slow process due to the size of the proteins and their concentration.

A role of every single PTM in cataractogenesis is to be elucidated. However it is likely that a contribution of a particular protein modification is not solely proportional to its concentration but is also a function of its type. Therefore, in spite of a relatively low concentration of pentosidine, this cross-link may play an important role in protein aggregation^38^.

Nevertheless, the inability to distinguish the emission of a relatively small concentration of pentosidine from the emission of the more abundant OH-Trp and ArgP modifications led us to neglect this component in the spectral decomposition.

It is important to note that fluorescence of “free” NFK, Kyn and OH-Kyn is heavily quenched, as evident by their picosecond lifetime constants (Supplemental Fig. S3f,h and j). The fluorescence lifetime constants of the respective amino residues were found to be much greater. This dramatic increase in the lifetime constants of Kyn and its derivatives suggests inhibition of quenching mechanisms operating in the excited state. Tuna *et al*.^39^ discussed two mechanisms of quenching of Kyn fluorescence by excited state proton transfer: 1) from the α-amino group to the keto group in the *trans*-conformation, and 2) from the ring-amino group to the keto group in the *cis*-conformation. The first mechanism obviously cannot operate in peptides and proteins because of the involvement of the α-amino group in the formation of peptide bond. Hence, only the second mechanism can operate. Deactivation of the S_1_ excited state of the Kyn residue with an average lifetime of 6.9 ns in the peptide suggests that the conformational equilibrium is shifted towards the *trans*-conformation in which the proton transfer cannot operate. The shorter average lifetimes of the NFK and OH-Kyn residues suggest that both of them can have *cis*- and *trans*-conformations in the model peptide.

A 7-fold increase in fluorescence quantum yield and ~0.3 ns average lifetime of Kyn fluorescence was reported upon its covalent conjugation to a protein^40^. However, this lifetime is significantly shorter than the 6.9 ns lifetime observed in the AA-Kyn-AA peptide. Our “global” lifetime analysis of fluorescence time-responses of human emulsified sample revealed a minor 0.25 ns lifetime component together with dominant 1.6 ns, 4.6 ns and 11.1 ns components (Supplemental Fig. S4). However, the spectrum associated with this minor component does not correlate with the emission spectrum of Kyn (lower *right panel, blue line*). Therefore, we suppose that that the major portion of non-Trp emission in the green-red spectral range is associated with the emission of modified Trp side chains rather than with the emission of covalently bound Kyn or its derivatives.

Thus, the emission from the primary products of Trp oxidation, OH-Trp and NFK, together with ArgP, dominates the fluorescence of crystallins, both in emulsified human samples and whole lenses upon the 317 nm excitation. The normalised cumulative sum of the above fluorescent PTMs shows a strong correlation with cataract grade and age. Millimolar concentrations and high luminosity of OH-Trp, NFK and ArgP make them easily detectable in the eye lens upon the red edge excitation, together with fluorescence of Trp. The 1–2% modification rate of Trp in the lens suggests that its concentration remains nearly constant. Thus, the normalisation of the fluorescence intensity of the fluorescent PTMs, against the intensity of Trp emission, gives a non-invasive, semi-quantitative cataract diagnostic, with greatly increased sensitivity over current techniques.

We and others^41^ identified a wide range of PTMs in crystallins and it is likely that all modifications may contribute to protein degradation. A question therefore arises - why does the fluorescent fraction, from all possible PTMs, correlate with cataract grade and age? While the significance of every PTM is not clearly understood, we hypothesise that the fluorescent fraction represents the total modification statistics. Moreover, some specific fluorescent PTMs has been shown to play important roles in cataractogenesis by changing the stability and interactions of proteins. Ghosh *et al*.^42^ demonstrated that the interaction between the *N*-terminal W60 and the core domain R123 plays a role in the monomeric interaction of α-crystallins involved in the formation of the α-crystallin oligomeric structure. The assembly of α-crystallins into large oligomeric complexes is believed to be central to the chaperone-like function of this protein^32^.

In addition, the formation of NFK from Trp is mediated by the interaction with singlet oxygen and, therefore, the concentration of NFK can be used as a marker of protein oxidative stress^43^. Moreover, NFK possesses photosensitizing properties, which make it the most biologically important photo-product of Trp because its formation facilitates the production of hydrogen peroxide in irradiated Trp solutions^44^,^45^,^46^. Hence, the formation of NFK can stimulate modifications of other side chains.

The abundant formation of ArgP in α-crystallins and in particular the conversion of Arg120 to ArgP in α-crystallin B is likely to affect its chaperone-like function. Vicart *et al*. identified the Arg120Gly mutation in α-crystallin B responsible for desmin-related myopathy in a French family^47^. It has also been shown that cataract and myopathy pathologies in αB-Arg120Gly knock-in mice share common mechanisms^48^ and mutation of the Arg120 residue in the human α-crystallin causes a partial loss of its chaperone-like activity^33^,^49^. All these findings strongly suggest that the concentration of Arg120 should negatively correlate with chaperone-like function of α-crystallin and hence may be used as a marker of cataract.

In summary, in this work we have developed a novel non-invasive method of semi-quantitative determination of the concentrations of fluorescent PTMs in the lens based on simultaneous measurements of the fluorescence emission spectra of Trp, its photo-degradation products and ArgP. We show that the fluorescence spectra of the individual fluorescent components normalised against Trp intensity enable determination of their relative concentration with an accuracy of 5–10%. This makes this method useful for cataract grading and for monitoring cataractogenesis over a period of time. The latter may help in elucidation of various metabolic and ambient factors contributing to cataractogenesis. The possibility to monitor changes in the lens structure at the molecular level may also facilitate the development of cataract medications, aimed at slowing down the cataractogenic processes and prolonging lens homeostasis.

## Methods

### Materials

Unless otherwise stated all chemicals were purchased from Sigma-Aldrich (UK) and used without further purification. ArgP was kindly gifted by Dr Nagaraj. NFK was purchased from Santa Cruz Biotechnology (USA). Pentosidine was purchased from Cambridge Bioscience (UK).

Porcine eyes were obtained from the local abattoir shortly after pig sacrifice. The eyes were dissected to extract the lenses taking care to avoid mechanical damage to the lens capsule. Spectral measurements were carried out using the Edinburgh Instruments in-house double-grating spectrometer FLS980 in a temperature controlled 1 cm fused silica cuvette (lenses) or semi-micro cuvette (solutions) in PBS (Dulbecco’s phosphate-buffered saline, modified) at 22 °C (this temperature retained uniform tissue viability and proved to give consistent results over ca. 24 h without visible change. The experimental geometry between excitation and emission channels was 90° with the samples aligned at 45°. Fluorescence spectra were measured using excitation and emission slits of 3 nm band-widths and corrected for spectral sensitivity of the emission tract.

Changes in the lens structure were induced by UV irradiation in a FLS980 single-grating spectrometer using 320 nm light with 20 nm bandwidth and 450 W Xenon arc lamp over a time-period of 24 h. The irradiation produced a rectangular shape volume of ~3 × 3 × 0.5 mm (X × Y × Z) size. The emission of irradiated lenses was measured from the irradiated spot on the lens surface. Fluorescence images of irradiated and normal porcine lenses and human donor lenses were taken by a Cannon 600D digital camera using the same exposure time and numerical aperture.

Human donor lenses were obtained from the Bristol eye bank (University of Bristol, UK) 2–3 days post-mortem. The lenses were shipped in MEM transport medium and transferred to PBS supplemented with Penicillin/Streptomycin antibiotics mixture (Penicillin 1000 units/mL and 1 mg/mL Streptomycin) and stored refrigerated at +4 °C. Lens samples were equilibrated for 30 min at 22 °C before measurements. The nuclei of all lenses exhibited yellow colouration of various intensities.

Post-operational phaco-emulsified lens samples obtained from Princess Alexandra Eye Pavilion (Edinburgh) were frozen at −20 °C before processing in the lab. Two clear emulsified lenses of 40 and 46 year old patients who had undergone a cosmetic surgery were used as control samples. Thawed samples of 20 ml volume were centrifuged at 14,000 × *g* for 1 h in 50 ml test tubes (Fisher Scientific, UK) in an Allegra X-30 centrifuge (Beckman Coulter, UK). Supernatants were concentrated 40-fold in Vivaspin 20 MWCO 10,000 centrifugal concentrators (Sigma-Aldrich). Pellets were re-suspended in 400 μl of PBS/8 M urea buffer and stored refrigerated at +4 °C.

Ac-AAWAA-NH_2_ (AAWAA) peptide of 95% purity was purchased from Cambridge Peptides (UK). A sample of the peptide solution in PBS (2 mg/ml) was irradiated in a semi-micro fused silica cuvette (Starna, UK) over 48 h in a single-grating FLS980 spectrometer by the 300 nm wavelength (20 nm bandwidth) light of ~7 mW intensity. The irradiated peptide was fractioned and analysed by ESI MS by the Almac analytical services group (Scotland). Fractionation of the peptide was carried out on an ÄKTA basic HPLC system using Luna 5 μm C18(2) 100 A (250 × 4.6 mm) column in acetonitrile: water gradient 10–80% over 30 minutes (mobile phase A = water 0.1% (v/v) TFA, mobile phase B = acetonitrile 0.1% (v/v) TFA). Lyophilised fractions were reconstituted in PBS for fluorescence analysis. LC-MS/MS Analysis was performed on a Bruker Daltonics microTOF mass spectrometer. Peptides were ionised using electrospray ionisation in positive ion mode.

Size exclusion chromatography of soluble crystallins was carried out in an ÄKTA FPLC instrument (GE Healthcare, GB) using Superdex 200 analytical column (GE Healthcare, GB) in PBS at 0.5 ml/min flow rate at ambient temperature.

### Fluorescence lifetime measurements

Time-Correlated Single Photon Counting (TCSPC) measurements were carried out in the reversed mode using 280 nm, 320 nm pulsed sub-nanosecond LED and 375 nm, 405 nm, 445 nm picosecond diode lasers (Edinburgh Instruments, Livingston, Scotland) at 10 Mhz repetition rate. Lifetime parameters were calculated by deconvolving experimental time-responses, *I(t*), from instrument response function *IRF(t*)


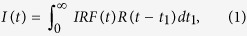


using a model:


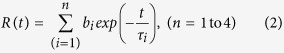


where *b*_*i*_ are pre-exponential coefficients and *τ*_*i*_ are lifetime parameters) independently or globally with ”linked” lifetime parameters. Average lifetimes <*τ*> were calculated as follows:


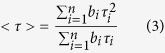


Decay associated spectra (DAS) of individual lifetime components were calculated from a series of fluorescence time-responses measured in equidistant-spaced 10 nm steps spectral points along the emission spectrum. The total number of counts under each time-response was normalised on the respective steady-state intensity. The data were evaluated globally with “linked” lifetime parameters and the calculated fractional contributions (*f*_*i*_ = *b*_*i*_ · *τ*_*i*_) of individual lifetime components were plotted as a function of wavelength.

### Confocal microscopy

Fluorescence intensity distribution along the optical axis of the lens were carried out in a F410 confocal microscope described elsewhere^50^. In brief, the instrument axially scans the focus of fibre-coupled CW 405 nm laser throughout the sample. The laser excited fluorescence is separated from the excitation by a dichroic mirror and spectrally filtered by an emission filter combination (cut-on 450 nm) was focused through a pinhole onto a photo-multiplier tube. The signal is further filtered and amplified by custom made electronics.

### Spectral decomposition

Decomposition of fluorescence spectra over a set of 6 spectral components was carried out by proprietary software written in Matlab and based on the Nelder-Mead minimisation algorithm using the following equation:


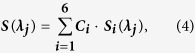


where *S(λ*_*j*_) and *S*_*i*_(λ_j_) are the a total and elementary spectra respectively measured in *λ*_j_ spectral points.

### Protein mass spectrometry

A human emulsified nuclear sample of nuclear cataract grade II (NC++) was separated into water-soluble and insoluble fractions by 1 h centrifugation at 14,000 × *g*. Protein samples were subjected to SDS-PAGE followed by band excision and peptide digestion. Peptides were ionized by nano-electrospray ionization at 2.1 kV using a stainless steel emitter with an internal diameter of 30 μm (Thermo Scientific, UK) and a capillary temperature of 250 °C. Tandem mass spectra were acquired using an LTQ-Orbitrap Velos mass spectrometer controlled by Xcalibur 2.1 software (Thermo Scientific) and operated in data-dependent acquisition mode. The Orbitrap was set to analyse the survey scans at 60,000 resolution (at m/z 400) in the mass range m/z 300 to 2000 and the top twenty multiply charged ions in each duty cycle selected for MS/MS in the LTQ linear ion trap. PTM abundance was estimated from spectral counting.

Only non-identifiable donor material was used and informed consent was obtained from all subjects. The study adhered to the tenets of the Helsinki Declaration and all experimental protocols were approved by the ethics committee of Heriot Watt University.

## Additional Information

**How to cite this article**: Gakamsky, A. *et al*. Tryptophan and Non-Tryptophan Fluorescence of the Eye Lens Proteins Provides Diagnostics of Cataract at the Molecular Level. *Sci. Rep.* **7**, 40375; doi: 10.1038/srep40375 (2017).

**Publisher's note:** Springer Nature remains neutral with regard to jurisdictional claims in published maps and institutional affiliations.

## Supplementary Material

Supplementary Information

## Figures and Tables

**Table 1:** PTM rates in crystallins of a human emulsified lens with nuclear cataract (NC++).

No	Protein	PTM type	Residue PTM rate (%)	Average PTM protein PTM rate (%)
1	α-Crystallin A	R68 → ArgP	1.3	6.1
		R112 → ArgP	0.7	
		R116 → ArgP	24.1	
		R1117 → ArgP	1.3	
		R119 → ArgP	41.0	
2	α-Crystallin B	W60 → NFK	1.3	0.75
		R22 → ArgP	1.6	10.7
		R107 → ArgP	1.5	
		R116 → ArgP	2.5	
		R120 → ArgP	97	
		R123→ArgP	24.5	
3	β-Crystallin A4	W54 → OH-Trp	0.5	0.4
		W149 → OH-Kyn	0.8	
		W179 → OH-Trp	1.3	
4	β-Crystallin B1	W216 → OH-Trp	7.3	1.3
		W237 → NFK	0.7	
		R233 → ArgP	1.7	0.3
5	β-Crystallin B2	W59 → OH-Kyn	2.5	3.9
		W85 → OH-Trp	2.1	
		W151→OH-Trp	9.1	
		R189 → ArgP	4.1	0.6
		R191 → ArgP	0.7	
