# Supplementary material for: Tryptophan and Non-Tryptophan Fluorescence of the Eye Lens Proteins Provides Diagnostics of Cataract at the Molecular Level
Source: Sci Rep. 2017 Jan 10;7:40375. doi: 10.1038/srep40375 (PMC5223181; doi:10.1038/srep40375)
Supplement: Supplementary Information [file srep40375-s1.pdf]

Tryptophan and Non-Tryptophan Fluorescence of the Eye Lens Proteins Provides Diagnostics of Cataract at the Molecular Level

Supplementary Material

Anna Gakamsky[1], Rory R. Duncan[2], Nicola M. Howarth[2], Baljean Dhillon[3], Kim K. Buttenschön[4], Daniel J. Daly[4] and Dmitry Gakamsky[2]

[1]Edinburgh Instruments, 2 Bain Square, Livingston, EH54 7DQ, UK; [2]Institute of Biological Chemistry, Biophysics and Bioengineering, School of Engineering and Physical Sciences, Heriot-Watt University, Edinburgh, EH14 6 UK; [3]Edinburgh Eye Pavilion, Chalmers Street, Edinburgh EH3 9HA, UK; [4]Lein Applied Diagnostics, Reading Enterprise Centre, Whiteknights Rd, Reading RG6 6BU, UK

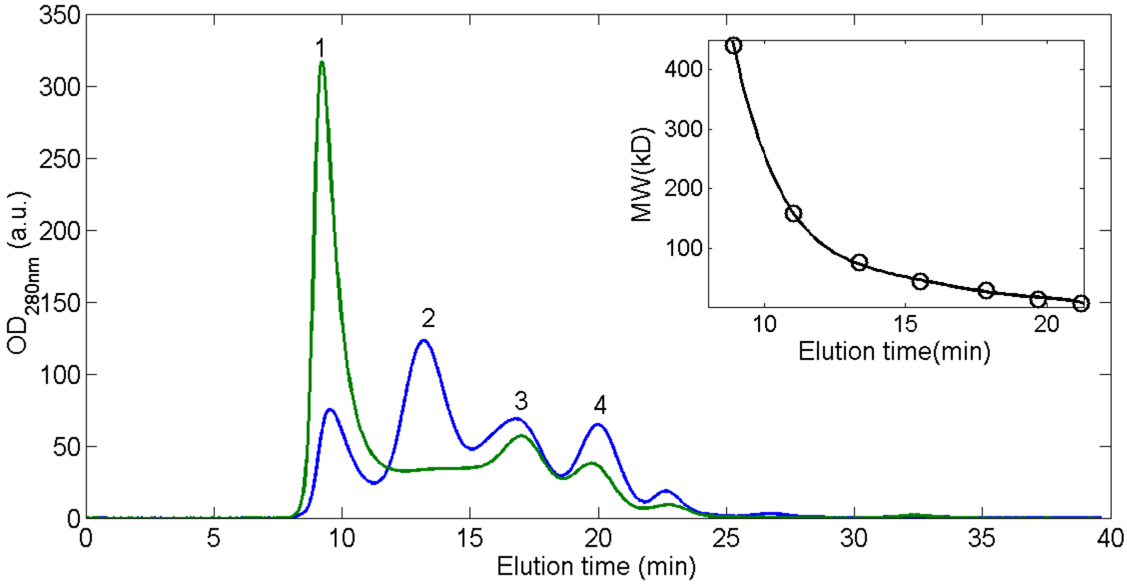

Figure S1. Size exclusion chromatograms of a control (blue) and UV-irradiated solubilised porcine eye lens samples (green) performed at 0.5ml/min flow rate in a Superdex 200 column (GE Healthcare). Inset: Size-exclusion column (Superdex 200) calibration curve. The data were obtained by applying samples of Aprotinin (6.5 kD; 21.21 min), Ribonuclease (13.7 kD; 19.68 min), Carbonic Anhydrase (29 kD; 17.86 min), Ovalbumin (44 kD; 15.50 min), Conalbumin (75 kD; 13.35 min), Aldolase (158 kD; 111.1 min) and Ferritin (440 kD; 8.91 min) in PBA to the column. The data were fitted by 5th degree polynomial function y([D])= $-10.557x^5 + 899.14x^4 - 30478x^3 + 5.1466e{+}005x^2 - 4.3436e{+}006x + 1.4768e{+}007$, ([x] min).

Supplemental Figure S2a

Irradiated sample

CRYAA_PIG (100%), 19,746.5 Da
Alpha-crystallin A chain OS=Sus scrofa GN=CRYAA PE=1 SV=1
19 exclusive unique peptides, 44 exclusive unique spectra, 429 total spectra, 159/173 amino acids (92% coverage)

```
MDIAIQHPWF KRALGPFYPS RLFDQFFGEG LFEYDLLPFL SSTISPYYRQ
SLFRTVLDSG VSEVRSDRDK FVIFLDVKHF SPEDLTVKVQ EDFVEIHGKH
NERQDDHGYI SREFHRRYRL PSNVDQSALS CSLSADGMLT FSGPKVPSGV
DAGHSERAIP VSREEKPSSA PTS
```

AA	Spectral counts		PMT rate (%)
M1	Unmodified<br>Acetyl (+42)<br>Oxidation (+16)	1<br>79<br>1	99
H7	Unmodified<br>Oxidation (+16)	54<br>1	1.8
W9	Unmodified<br>Oxidation (+16) Trp => OH-Trp<br>Dioxidation (+32) Trp=> NFK<br>(+4) Trp=> Kyn	46<br>2<br>5<br>2	16.4
H79	Unmodified<br>Oxidation (+16)<br>Acetyl (+42)	85<br>1<br>1	2.3
H97	Unmodified<br>Oxidation (+16)	62<br>2	3.1
H104	Unmodified<br>Gln=>pyro-Glu (+17)	6<br>1	14.3
C131	Unmodfied<br>Carbabidolethyl (+57)	0<br>35	100
M138	Unmodified<br>Oxidation (+16)	25<br>6	19.4
H154	Unmodified<br>Oxidation (+16)	20<br>1	4.8

Control sample

CRYAA_PIG (100%), 19,746.5 Da
Alpha-crystallin A chain OS=Sus scrofa GN=CRYAA PE=1 SV=1
21 exclusive unique peptides, 42 exclusive unique spectra, 643 total spectra, 163/173 amino acids (94% coverage)

```
MDIAIQHPWF KRALGPFYPS RLFDQFFGEG LFEYDLLPFL SSTISPYYRQ
SLFRTVLDSG VSEVRSDRDK FVIFLDVKHF SPEDLTVKVQ EDFVEIHGKH
NERQDDHGYI SREFHRRYRL PSNVDQSALS CSLSADGMLT FSGPKVPSGV
DAGHSERAIP VSREEKPSSA PTS
```

AA	Spectral counts		PTM rate (%)
M1	Unmodified<br>Acetyl (+42)<br>Oxidation (+16)	1<br>79<br>1	98.8
H7	Unmodified<br>Oxidation (+16)	81<br>0	0
W9	Unmodified<br>Oxidation (+16) Trp => OH-Trp<br>Dioxidation (+32) Trp=> NFK<br>(+4) Trp=> Kyn	77<br>1<br>5<br>1	8.3
H79	Unmodified<br>Oxidation (+16)<br>Acetyl (+42)	91<br>0<br>1	1.1
H97	Unmodified<br>Oxidation (+16)	114<br>0	0
H104	Unmodified<br>Gln=>pyro-Glu (_17)	12<br>7	36.8
C131	Unmodfied<br>Carbabidolethyl (+57)	0<br>66	100
M138	Unmodified<br>Oxidation (+16)	59<br>7	10.1
H154	Unmodified<br>Oxidation (+16)	51<br>0	0

Supplemental Figure S2b

Irradiated sample

B1Q039_PIG (100%), 20,129.4 Da
CRYAB OS=Sus scrofa GN=CRYAB PE=3 SV=1
17 exclusive unique peptides, 43 exclusive unique spectra, 230 total spectra, 154/175 amino acids (88% coverage)

```
MDIAIHHPWI RRPFFPFHSP SRLFDQFFGE HLLESDLFPA STSLSPFYFR
PPSFLRAPSW IDTGLSEMRL EKDRFSVNLD VKHFSPEELK VKVLGDVIEV
HGKHEERQDE HGFISREFHR KYRIPADVDP LTITSSLSSD GVLTVNGPRR
QASGPERTIP ITREEKPAVT AAPKK
```

AA	Spectral counts		PMT rate (%)
M1	Unmodified	0	100
	Acetyl (+42)	16	
	Oxidation (+16)	1	
H6	Unmodified	17	5.6%
	Oxidation (+16)	1	
H7	Unmodified	17	5.6
	Oxidation (+16)	1	
W9	Unmodified	13	27.8
	Dioxidation (+32) Trp=> NFK	4	
	(+4) Trp=> Kyn	1	
W60	Unmodified	8	60
	Oxidation (+16)	5	
	Dioxidation (+32) Trp=> NFK	6	
	(+4) Trp=> Kyn	1	
M68	Unmodified	11	45
	Oxidation(+16)	9	
V91	Unmodified	0	100
	Acetyl (+42)	2	
Q108	Unmodified	3	66.7
	Gln=>pyro-Glu (+17)	6	

Control sample

B1Q039_PIG (100%), 20,129.4 Da
CRYAB OS=Sus scrofa GN=CRYAB PE=3 SV=1
22 exclusive unique peptides, 47 exclusive unique spectra, 380 total spectra, 161/175 amino acids (92% coverage)

```
MDIAIHHPWI RRPFFPFHSP SRLFDQFFGE HLLESDLFPA STSLSPFYFR
PPSFLRAPSW IDTGLSEMRL EKDRFSVNLD VKHFSPEELK VKVLGDVIEV
HGKHEERQDE HGFISREFHR KYRIPADVDP LTITSSLSSD GVLTVNGPRR
QASGPERTIP ITREEKPAVT AAPKK
```

AA	Spectral counts		PMT rate (%)
M1	Unmodified	0	100
	Acetyl (+42)	28	
	Oxidation (+16)	1	
H6	Unmodified	31	0
	Oxidation (+16)	0	
H7	Unmodified	31	0
	Oxidation (+16)	0	
W9	Unmodified	29	6.5
	Dioxidation (+32) Trp=> NFK	1	
	(+4) Trp=> Kyn	1	
W60	Unmodified	23	8
	Oxidation (+16)	0	
	Dioxidation (+32) Trp=> NFK	2	
	(+4) Trp=> Kyn	0	
M68	Unmodified	21	16
	Oxidation(+16)	4	
V91	Unmodified	2	50
	Acetyl (+42)	2	
Q108	Unmodified	3	66.7
	Gln=>pyro-Glu (_17)	6	

Supplemental Figure S2c

CRBB1_PIG (100%), 27,912.3 Da
Beta-crystallin B1 OS=Sus scrofa GN=CRYBB1 PE=2 SV=1
18 exclusive unique peptides, 35 exclusive unique spectra, 97 total spectra, 214/249 amino acids (86% coverage)

```
MSQPAVKASA TAAVNPGPDG KGKGAPPPGP APGSGPAQAP AQPMPAAKGD
LPPGSYKLVV FEQENFQGRR VEFSGECLNL GDRGFDRVRS IIVTSGPWVA
FEQSNFRGEM FILEKGEYPR WDTWSSSYRS DRLMSFRPIR MDAQEHKLCL
FEGANFKGNT MEIQEDDVPS LWVYGFCDRV GSVRVSSGTW VGYQYPGYRG
YQYLLEPGDF RHWNDWGAFQ PQMQAVRRLR DRQWHREGCF PVLAAEPPK
```

AA	Spectral counts		PMT rate (%)
M54	Unmodified<br>Oxidation (+16)	4<br>4	50
C77	Unmodified<br>Carbamidomethyl (+57)	0<br>6	100
S90	Unmodified<br>Acetyl (+42)	14<br>1	6.7
W98	Unmodified<br>Oxidation (+16) Trp=>OH-Trp<br>Dioxidation (+32) Trp=> NFK	12<br>3<br>0	20
M110	Unmodified<br>Oxidation (+16)	2<br>1	33.3
W121	Unmodified<br>Oxidation (+16) Trp=>OH-Trp<br>Dioxidation (+32) Trp=> NFK	3<br>1<br>0	25
W124	Unmodified<br>Oxidation (+16) Trp=>OH-Trp	3<br>1	25
C149	Unmodified<br>Acetyl (+42)	0<br>12	100
M161	Unmodified<br>Oxidation (+16)	2<br>2	50
C177	Unmodified<br>Carbamidomethyl	0<br>7	100
W190	Unmodified<br>Oxidation (+16) Trp=>OH-Trp	2<br>2	50
W216	Unmodified<br>Oxidation (+16)	5<br>1	16.7
M233	Unmodified<br>Oxidation (+16)	4<br>2	33.3
C249	Unmodified<br>Carbamidomethyl (+57)	0<br>3	100

CRBB1_PIG (100%), 27,912.3 Da
Beta-crystallin B1 OS=Sus scrofa GN=CRYBB1 PE=2 SV=1
16 exclusive unique peptides, 26 exclusive unique spectra, 83 total spectra, 201/249 amino acids (81% coverage)

```
MSQPAVKASA TAAVNPGPDG KGKGAPPPGP APGSGPAQAP AQPMPAAKGD
LPPGSYKLVV FEQENFQGRR VEFSGECLNL GDRGFDRVRS IIVTSGPWVA
FEQSNFRGEM FILEKGEYPR WDTWSSSYRS DRLMSFRPIR MDAQEHKLCL
FEGANFKGNT MEIQEDDVPS LWVYGFCDRV GSVRVSSGTW VGYQYPGYRG
YQYLLEPGDF RHWNDWGAFQ PQMQAVRRLR DRQWHREGCF PVLAAEPPK
```

AA	Spectral counts		PMT rate (%)
M54	Unmodified<br>Oxidation (+16)	6<br>0	0
C77	Unmodified<br>Carbamidomethyl (+57)	0<br>14	100
S90	Unmodified<br>Acetyl (+42)	14<br>1	6.7
W98	Unmodified<br>Oxidation (+16) Trp=>OH-Trp<br>Dioxidation (+32) Trp=> NFK	12<br>0<br>1	7.7
M110	Unmodified<br>Oxidation (+16)	1<br>0	0
W121	Unmodified<br>Oxidation (+16) Trp=>OH-Trp<br>Dioxidation (+32) Trp=> NFK	3<br>1<br>1	40
W124	Unmodified<br>Oxidation (+16) Trp=>OH-Trp	3<br>0	0
C149	Unmodified<br>Acetyl (+42)	0<br>12	100
M161	Unmodified<br>Oxidation (+16)	4<br>2	33.3
C177	Unmodified<br>Carbamidomethyl	0<br>2	100
W190	Unmodified<br>Oxidation (+16) Trp=>OH-Trp	1<br>0	0
W216	Unmodified<br>Oxidation (+16) Trp=>OH-Trp	6<br>0	0
M233	Unmodified<br>Oxidation (+16)	4<br>2	33.3
C249	Unmodified<br>Carbamidomethyl (+57)	0<br>5	

Supplemental Figure S2d

Irradiated sample

F1RG87_PIG (100%), 23,336.5 Da
Uncharacterized protein OS=Sus scrofa GN=CRYBB2 PE=4 SV=1
19 exclusive unique peptides, 34 exclusive unique spectra, 67 total spectra, 161/205 amino acids (79% coverage)

```
MASDHQTQAG KPQPLNPKII IFEQENFQGH SHELNGPCPN LKETGVEKAG
SVLVQAGPWV GYEQANCKGE QFVFEKGEYP RWDSWTSSRR TDSLSSLRPI
KVDSQEHKII LYENPNFTGK KMEVIDDDVP SFHAHGYQEK VSSVRVQSGT
WVGYQYPGYR GLQYLLEKGD YKDSGDFGAP QPQVQSVRRI RDMQWHQRGA
FHPSN
```

AA	Spectral counts		PMT rate (%)
A2	Unmodified<br>Acetyl (+42)	0<br>8	100
C42	No coverage		—
C67	Unmodified<br>Carbamidomethyl (+57)	0<br>3	100
W82	Unmodified<br>Oxidation (+16)	3<br>1	25
M122	Unmodified<br>Oxidation (+16)	4<br>4	50
W151	Unmodified<br>Oxidation (+16) Trp=>OH-Trp<br>Dioxidation (+32) Trp=> NFK	4<br>1<br>1	33.3

Control sample

F1RG87_PIG (100%), 23,336.5 Da
Uncharacterized protein OS=Sus scrofa GN=CRYBB2 PE=4 SV=1
16 exclusive unique peptides, 22 exclusive unique spectra, 27 total spectra, 172/205 amino acids (84% coverage)

```
MASDHQTQAG KPQPLNPKII IFEQENFQGH SHELNGPCPN LKETGVEKAG
SVLVQAGPWV GYEQANCKGE QFVFEKGEYP RWDSWTSSRR TDSLSSLRPI
KVDSQEHKII LYENPNFTGK KMEVIDDDVP SFHAHGYQEK VSSVRVQSGT
WVGYQYPGYR GLQYLLEKGD YKDSGDFGAP QPQVQSVRRI RDMQWHQRGA
FHPSN
```

AA	Spectral counts		PMT rate (%)
A2	Unmodified<br>Acetyl (+42)	0<br>8	100
C42	Unmodified<br>Carbamidomethyl	0<br>1	100
C67	Unmodified<br>Carbamidomethyl (+57)	0<br>1	100
W82	Unmodified<br>Oxidation (+16)	1<br>0	0
M122	Unmodified<br>Oxidation (+16)	4<br>0	0
W151	Unmodified<br>Oxidation (+16) Trp=>OH-Trp<br>Dioxidation (+32) Trp=> NFK	1<br>1<br>0	50

Supplemental Figure S2e

Irradiated sample

F2Z4Y7_PIG (100%), 24,256.0 Da
Uncharacterized protein OS=Sus scrofa GN=CRYBB3 PE=4 SV=1
16 exclusive unique peptides, 31 exclusive unique spectra, 61 total spectra, 173/211 amino acids (82% coverage)

```
MAEQHGTPEQ AAAGKSHGGL GGGYKVIVYE MENFQGKRCE LSAECPNLTE
SLLEKVGSIQ VESGPWLAFE RRAFRGEQFV LEKGDYPRWD AWSNSHHSDS
LLSLRPLHID GPDHKLHLFE NPAFGGRKME IVDDDVPSLW AHGFQDRVAS
IRAINGTWVG YEFPGYRGRQ YVFERGEYRH WNEWDANQPQ LQSVRRIRDQ
KWHKRGCFLS S
```

AA	Spectral counts		PMT rate (%)
A2	Unmodified<br>Acetyl (+42)	0<br>2	100
M31	Unmodified<br>Oxidation (+16)	3<br>1	25
C39	Unmodified<br>Carbamidomethyl (+57)	0<br>5	100
C45	Unmodified<br>Carbamidomethyl (+57)	0<br>5	100
W66	Unmodified<br>Oxidation (+16)	16<br>1	6.3
H117	Unmodified<br>Oxidation (+16)	4<br>1	20
M129	Unmodified<br>Oxidation (+16)	4<br>2	33.3
W158	Unmodified<br>Oxidation (+16) Trp=>OH-Trp	7<br>1	12.5

Control sample

F2Z4Y7_PIG (100%), 24,256.0 Da
Uncharacterized protein OS=Sus scrofa GN=CRYBB3 PE=4 SV=1
15 exclusive unique peptides, 23 exclusive unique spectra, 47 total spectra, 161/211 amino acids (76% coverage)

```
MAEQHGTPEQ AAAGKSHGGL GGGYKVIVYE MENFQGKRCE LSAECPNLTE
SLLEKVGSIQ VESGPWLAFE RRAFRGEQFV LEKGDYPRWD AWSNSHHSDS
LLSLRPLHID GPDHKLHLFE NPAFGGRKME IVDDDVPSLW AHGFQDRVAS
IRAINGTWVG YEFPGYRGRQ YVFERGEYRH WNEWDANQPQ LQSVRRIRDQ
KWHKRGCFLS S
```

AA	Spectral counts		PMT rate (%)
A2	Unmodified<br>Acetyl (+42)	0<br>2	100
M31	Unmodified<br>Oxidation (+16)	2<br>1	33.3
C39	Unmodified<br>Carbamidomethyl (+57)	0<br>3	100
C45	Unmodified<br>Carbamidomethyl (+57)	0<br>53	100
W66	Unmodified<br>Oxidation (+16)	11<br>0	0
H117	Unmodified<br>Oxidation (+16)	4<br>0	0
M129	Unmodified<br>Oxidation (+16)	4<br>0	0
W158	Unmodified<br>Oxidation (+16) Trp=>OH-Trp	8<br>0	0

Supplemental Figure S2f

Irradiated sample

F1RN90_PIG (100%), 25,167.9 Da
Uncharacterized protein OS=Sus scrofa GN=Ssc.22384 PE=4 SV=1
16 exclusive unique peptides, 33 exclusive unique spectra, 64 total spectra, 197/215 amino acids (92% coverage)

```
METQTLQQEL ESLPTTKMAQ TNPMPGSVGP WKITIYDQEN FQGKRMEFTS
SCPNVSERSF DNVRSLKVEC GAWVGYEHTS FCGQQFVLER GEYPRWDAWS
GSNAYHIERL MSFRPICSAN HKESKITIFE KENFIGRQWE ICDDYPSLQA
MGWFNNEVGS MKIQCGAWVC YQYPGYRGYQ YILECDHHGG DYKHWREWGS
HAQTSQIQSI RRIQQ
```

AA	Spectral counts		PMT rate (%)
M1	Unmodified<br>Acetyl (+42)<br>Oxidation (+16)	0<br>3<br>1	100
M18	Unmodified<br>Oxidation (+16)	4<br>5	55.6
M24	Unmodified<br>Oxidation (+16)	4<br>5	55.6
M56	Unmodified<br>Oxidation (+16)	4<br>4	50
C62	Unmodified<br>Carbamidomethyl (+57)	0<br>8	100
C70	Unmodified<br>Carbamidomethyl (+57)	0<br>4	100
C82	Unmodified<br>Carbamidomethyl (+57)	0<br>1	100
W96	Unmodified<br>Oxidation (+16) Trp=>OH-Trp	7<br>2	22.2
H106	Unmodified<br>Oxidation (+16)	7<br>2	22.2
M111	Unmodified<br>Oxidation (+16)	4<br>0	0
C117	Unmodified<br>Carbamidomethyl (+57)	0<br>1	100
C142	Unmodified<br>Carbamidomethyl (+57)	0<br>2	100
C165	Unmodified<br>Carbamidomethyl (+57)	0<br>6	100
C170	Unmodified<br>Carbamidomethyl (+57)	0<br>6	100
C185	Unmodified<br>Carbamidomethyl (+57)	0<br>4	100
W198	Unmodified<br>Oxidation (+16) Trp=>OH-Trp<br>Dioxidation (+32) Trp=>NFK	4<br>1<br>1	20

Control sample

F1RN90_PIG (100%), 25,167.9 Da
Uncharacterized protein OS=Sus scrofa GN=Ssc.22384 PE=4 SV=1
11 exclusive unique peptides, 15 exclusive unique spectra, 20 total spectra, 171/215 amino acids (80% coverage)

```
METQTLQQEL ESLPTTKMAQ TNPMPGSVGP WKITIYDQEN FQGKRMEFTS
SCPNVSERSF DNVRSLKVEC GAWVGYEHTS FCGQQFVLER GEYPRWDAWS
GSNAYHIERL MSFRPICSAN HKESKITIFE KENFIGRQWE ICDDYPSLQA
MGWFNNEVGS MKIQCGAWVC YQYPGYRGYQ YILECDHHGG DYKHWREWGS
HAQTSQIQSI RRIQQ
```

AA	Spectral counts		PMT rate (%)
M1	Unmodified<br>Acetyl (+42)<br>Oxidation (+16)	0<br>1<br>0	100
M18	Unmodified<br>Oxidation (+16)	2<br>1	33.3
M24	Unmodified<br>Oxidation (+16)	2<br>1	33.3
M56	Unmodified<br>Oxidation (+16)	2<br>0	0
C62	Unmodified<br>Carbamidomethyl (+57)	0<br>8	100
C70	Unmodified<br>Carbamidomethyl (+57)	0<br>2	100
C82	Unmodified<br>Carbamidomethyl (+57)	0<br>1	100
W96	Unmodified<br>Oxidation (+16) Trp=>OH-Trp	3<br>0	0
H106	Unmodified<br>Oxidation (+16)	3<br>0	0
M111	Unmodified<br>Oxidation (+16)	4<br>0	0
C117	No coverage		—
C142	Unmodified<br>Carbamidomethyl (+57)	1<br>2	66.7
C165	Unmodified<br>Carbamidomethyl (+57)	0<br>1	100
C170	Unmodified<br>Carbamidomethyl (+57)	0<br>1	100
C185	Unmodified<br>Carbamidomethyl (+57)	0<br>1	100
W198	Unmodified<br>Oxidation (+16) Trp=>OH-Trp<br>Dioxidation (+32) Trp=>NFK	1<br>0<br>0	0

Supplemental Figure S2g

Irradiated sample

F2Z4Y6_PIG (100%), 18,683.1 Da
Uncharacterized protein (Fragment) OS=Sus scrofa GN=CRYBA4 PE=4 SV=2
8 exclusive unique peptides, 15 exclusive unique spectra, 32 total spectra, 126/164 amino acids (77% coverage)

```
IVVWDEEGFQ GRRHEFTAEC PSVLELGFET VRSLKVLSGA WVGFEHAGFQ
GQQYVLERGE YPSWDAWSGN TAYPAERLTS FRPVACANHR DSRLTIFEQE
NFLGKKGELS DDYPSLQAMG WDGNEVGSFH VHSGAWVCSQ FPGYRGFQYV
LESGDYKHFR EWGS
```

AA	Spectral counts		PMT rate (%)
R13	Unmodified<br>Acetyl (+42)	0<br>1	100
C20	Unmodified<br>Carbamidomethyl (+57)	0<br>9	100
W41	Unmodified<br>Oxidation (+16) Trp=>OH-Trp	6<br>1	14.3
W63	Unmodified<br>Oxidation (+16) Trp=>OH-Trp	2<br>2	50
C138	Unmodified<br>Carbamidomethyl (+57)	0<br>1	100

Control sample

F2Z4Y6_PIG (100%), 18,683.1 Da
Uncharacterized protein (Fragment) OS=Sus scrofa GN=CRYBA4 PE=4 SV=2
6 exclusive unique peptides, 9 exclusive unique spectra, 17 total spectra, 86/164 amino acids (52% coverage)

```
IVVWDEEGFQ GRRHEFTAEC PSVLELGFET VRSLKVLSGA WVGFEHAGFQ
GQQYVLERGE YPSWDAWSGN TAYPAERLTS FRPVACANHR DSRLTIFEQE
NFLGKKGELS DDYPSLQAMG WDGNEVGSFH VHSGAWVCSQ FPGYRGFQYV
LESGDYKHFR EWGS
```

AA	Spectral counts		PMT rate (%)
R13	Unmodified<br>Acetyl (+42)	2<br>0	0
C20	Unmodified<br>Carbamidomethyl (+57)	0<br>4	100
W41	Unmodified<br>Oxidation (+16) Trp=>OH-Trp	2<br>0	0
W63	Unmodified<br>Oxidation (+16) Trp=>OH-Trp	1<br>0	0
C138	No coverage		---

Figure S2. PTM rates in the UV-irradiated and control samples of porcine eye lens proteins collected in the first fraction (peak 1 in Fig. S1) of size exclusion chromatography. The modification rates were estimated from spectral counting.

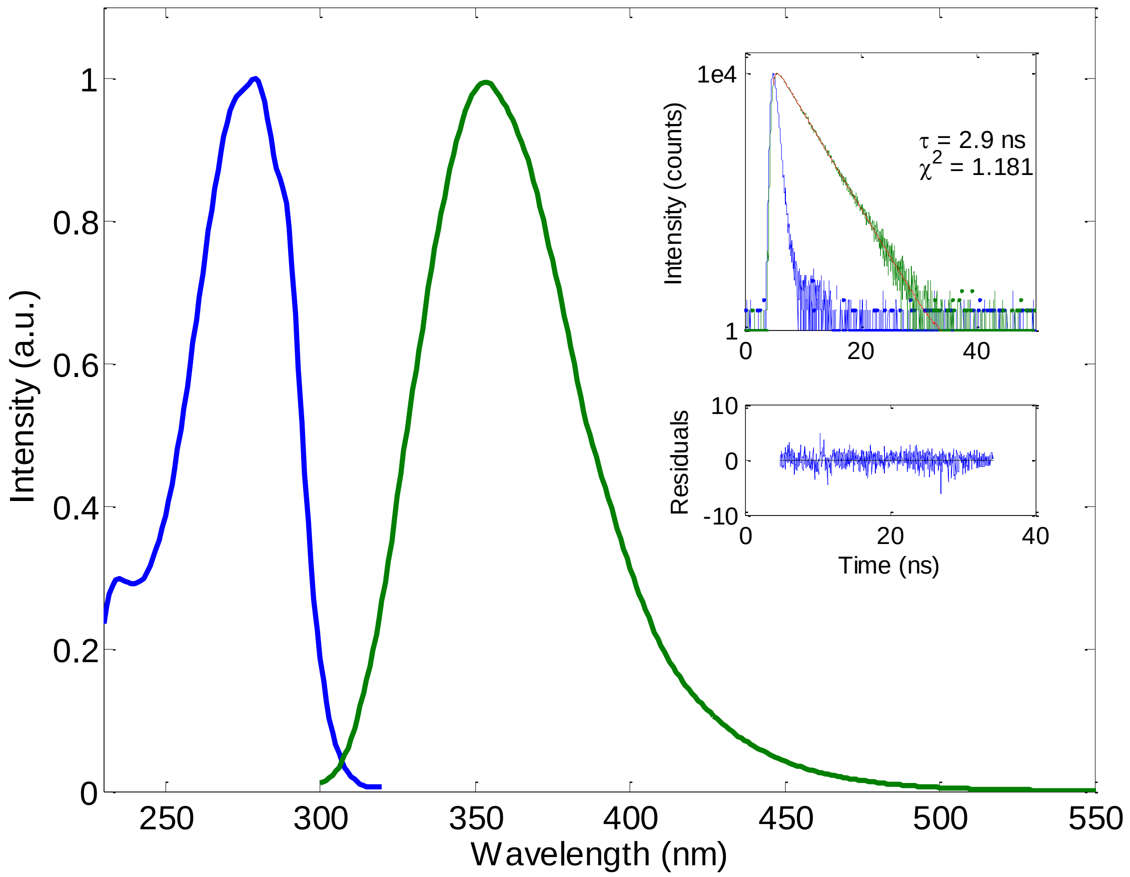

Figure S3a. Fluorescence excitation (blue) and emission (green) spectra and lifetime measurements (inset) of NATA in PBS. The fluorescence time-response was measured at 280 nm upon 350 nm excitation and fitted by a mono-exponential model (τ = 2.9 ns).

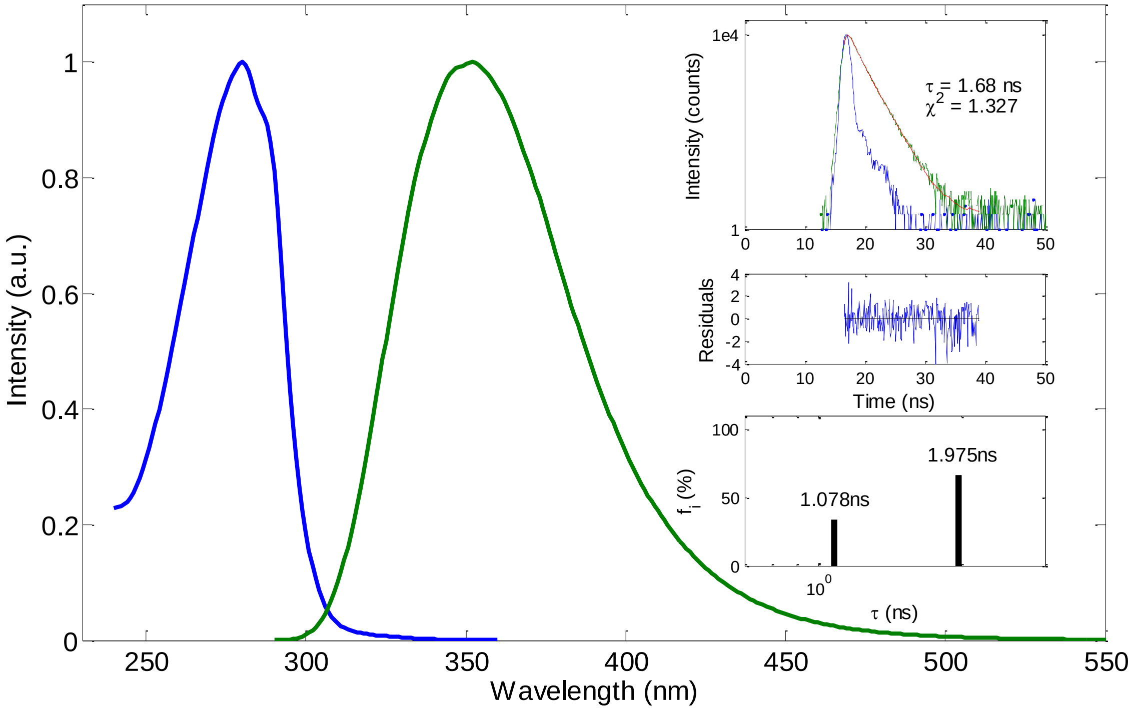

Figure S3b. Fluorescence excitation (blue) and emission (green) spectra and lifetime measurements (inset) of Ac-AAWAA-NH_2 peptide in PBS. The fluorescence time-response was measured at 350 nm upon 280 nm excitation and fitted by a double-exponential model (τ_1 = 1.1 ns (33.4%) and τ_2 = 2.0ns (66.6%); average lifetime τ = 1.7 ns).

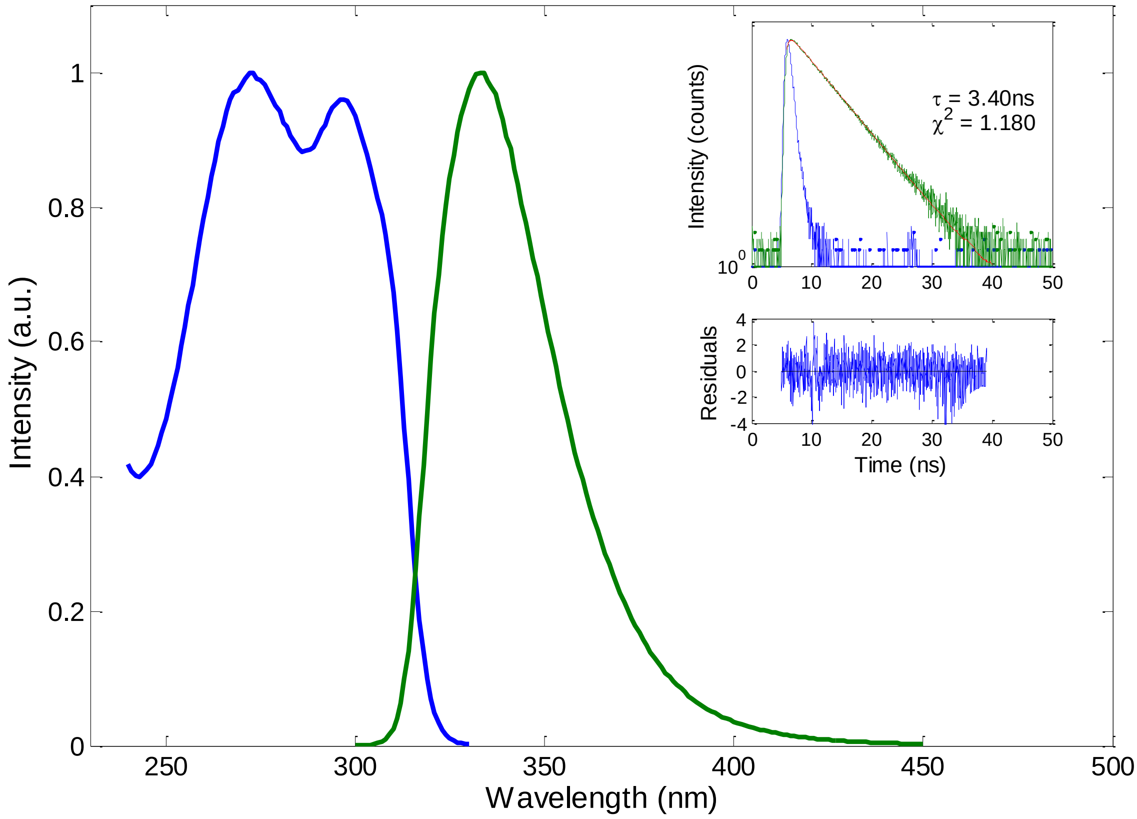

Figure S3c. Fluorescence excitation (blue) and emission (green) spectra and lifetime measurements (inset) of OH-Trp in PBS. The fluorescence time-response was measured at 350 nm upon 280 nm excitation and fitted by a single-exponential model (τ = 3.4 ns).

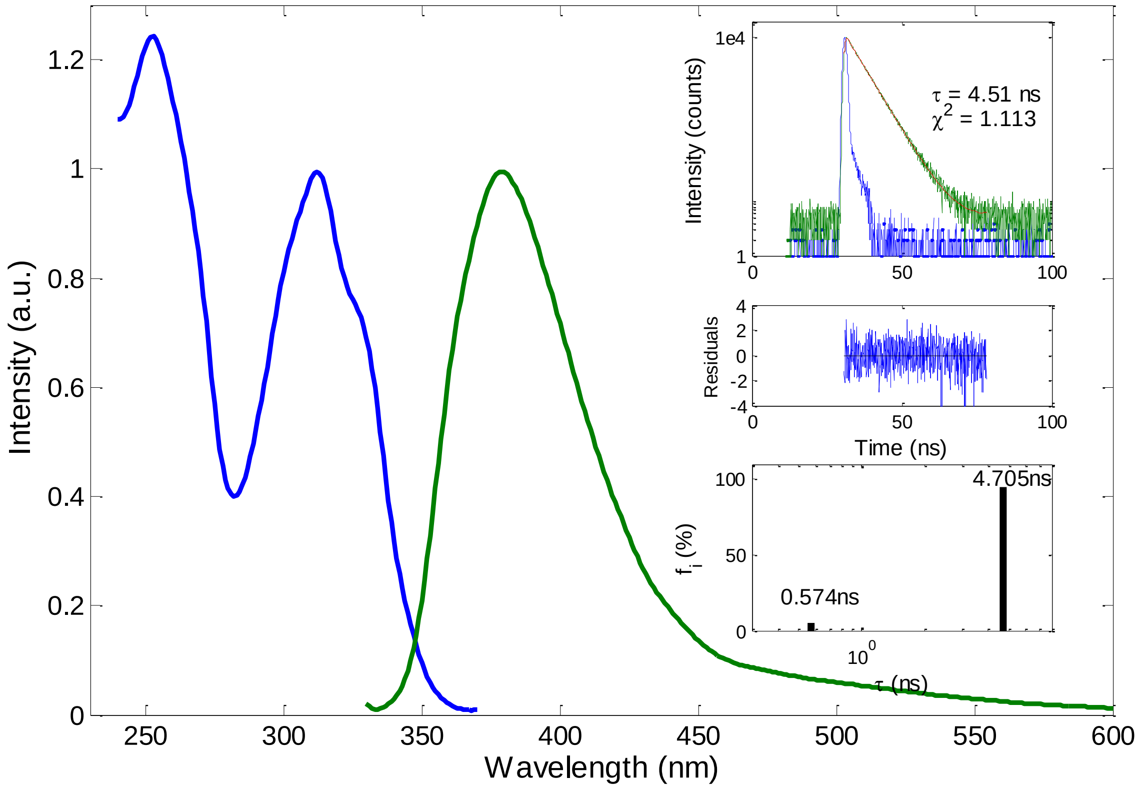

Figure S3d. Fluorescence excitation (blue) and emission (green) spectra and lifetime measurements (inset) of Ac-AA-OH-Trp-AA-NH_2 in PBS. The fluorescence time-response was measured at 380 nm upon 317 nm excitation and fitted by a double-exponential model (τ_1 = 0.6 ns (4.8%) and τ_2 = 4.7 ns (95.2%); average lifetime τ =4.5 ns).

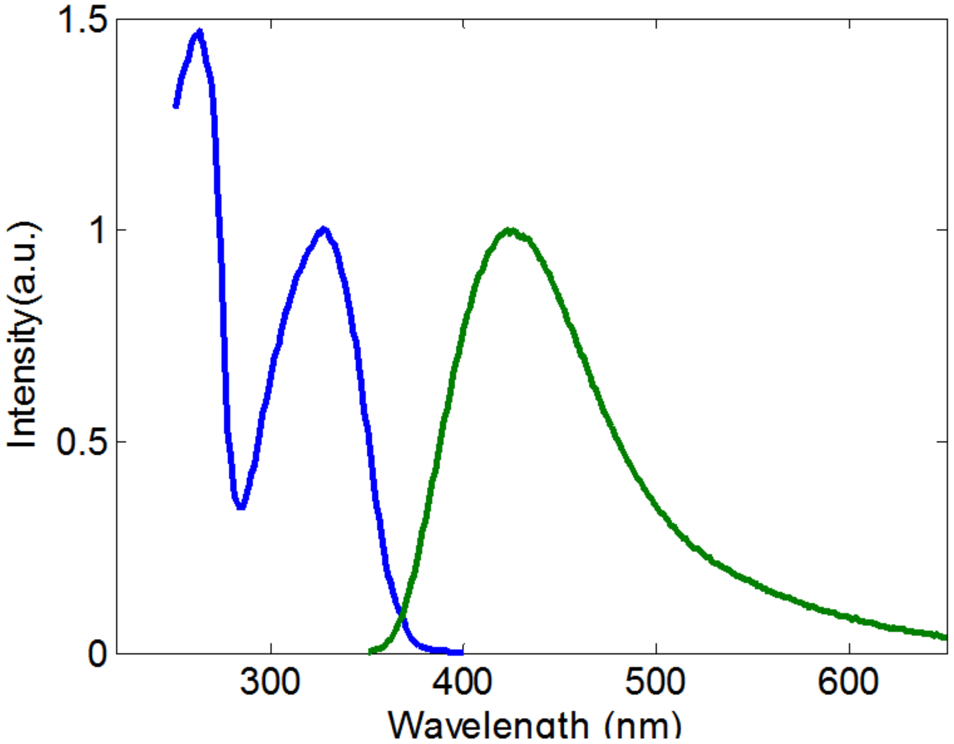

Figure S3e. Fluorescence excitation (blue) and emission (green) spectra of NFK in PBS.

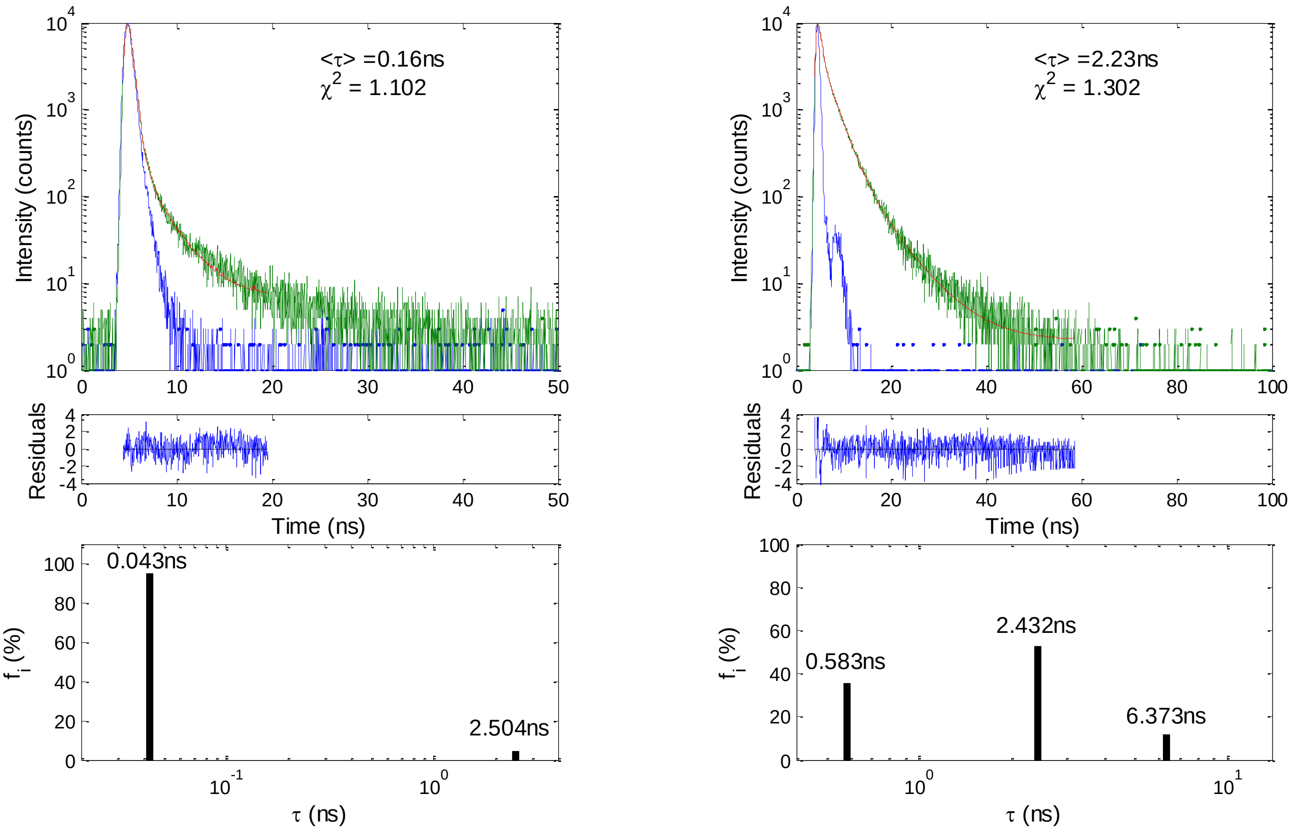

Figure S3f. Fluorescence lifetime measurements of NFK at 420 nm upon 317 nm excitation (left) and its residue in Ac-AA-NFK-AA-NH_2 at 420 nm upon 372 nm excitation (right). The data were fitted by a double-exponential model (τ_1 = 43 ps (95.4%) and τ_2 = 2.5 ns (4.6%), average lifetime 0.17 ns) or a 3-exponential model (τ_1=0.6 ns (35.5), τ_2=2.4 ns (52.8%) and τ_3 = 6.4 ns (11.7%), average lifetime 2.23 ns) respectively.

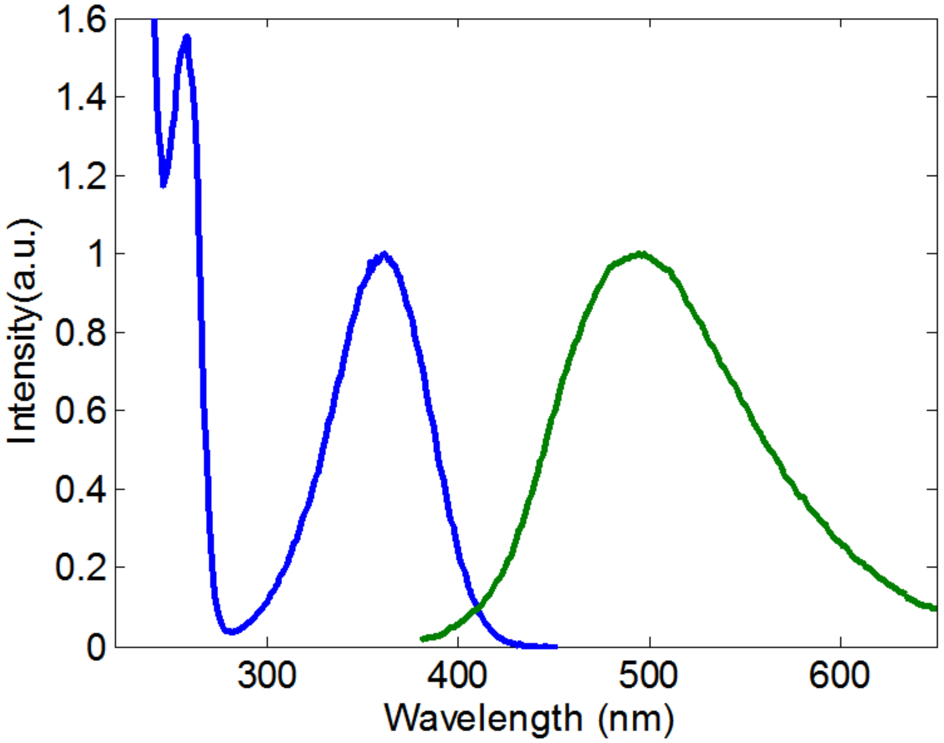

Figure S3g. Fluorescence excitation (blue) and emission (green) spectra of Kyn in PBS.

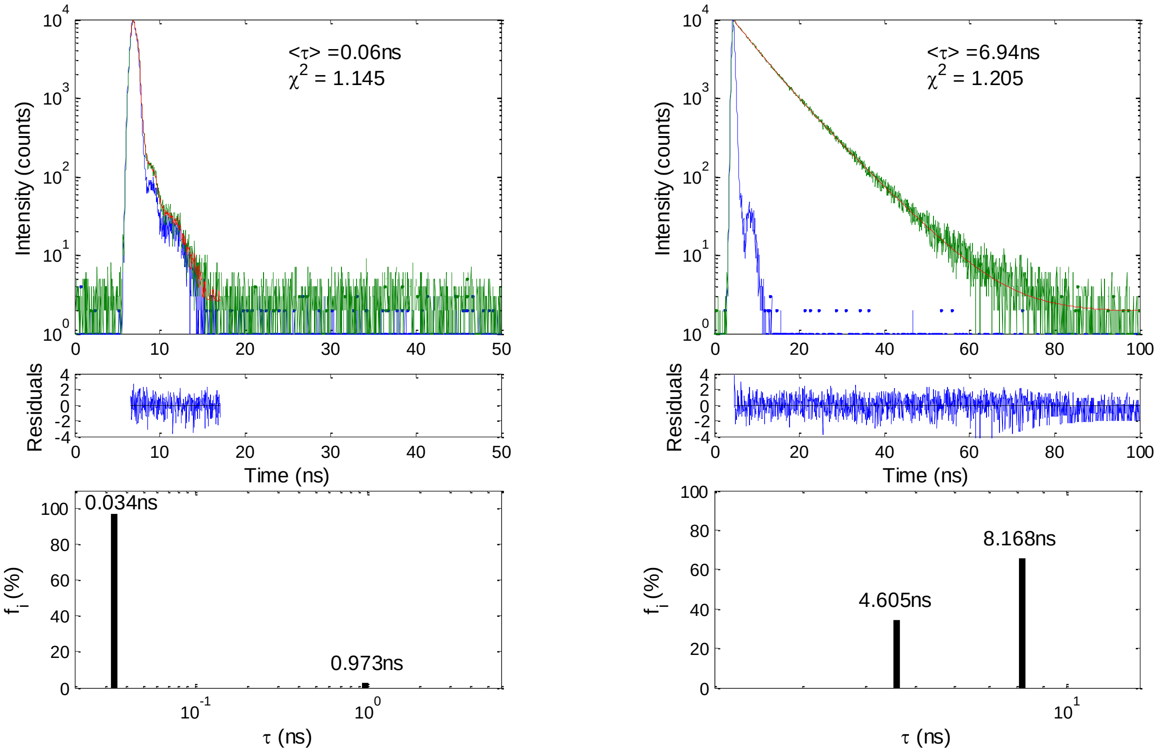

Figure S3h. Fluorescence lifetime measurements of Kyn 500nm (left) and its residue in Ac-AA-Kyn-AA-NH_2 at 540nm upon 372nm excitation (right). The data were fitted by a double- exponential models, Kyn (τ_1=34 ±15ps (95.4%) and τ_2=0.97ns (4.6%), average lifetime 60ps), and Ac-AA-Kyn-AA-NH_2 (τ_1= 4.6ns (34.6%) and τ_1=8.2nsns (65.4%), average lifetime 6.9ns).

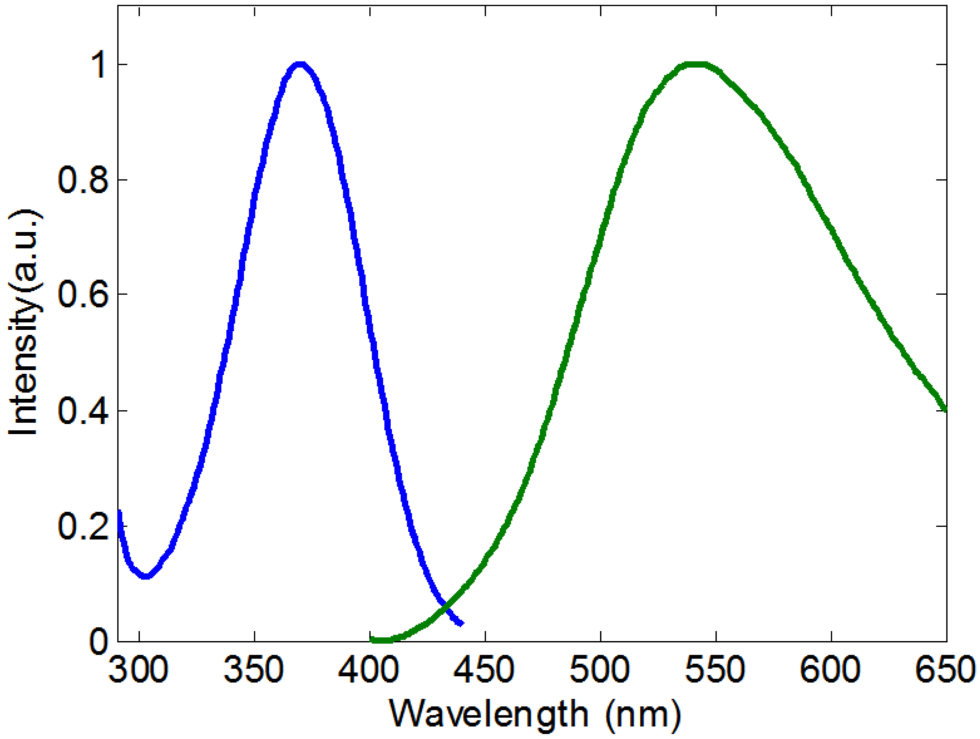

Figure S3i. Fluorescence excitation (blue) and emission (green) spectra of OH-Kyn in PBS.

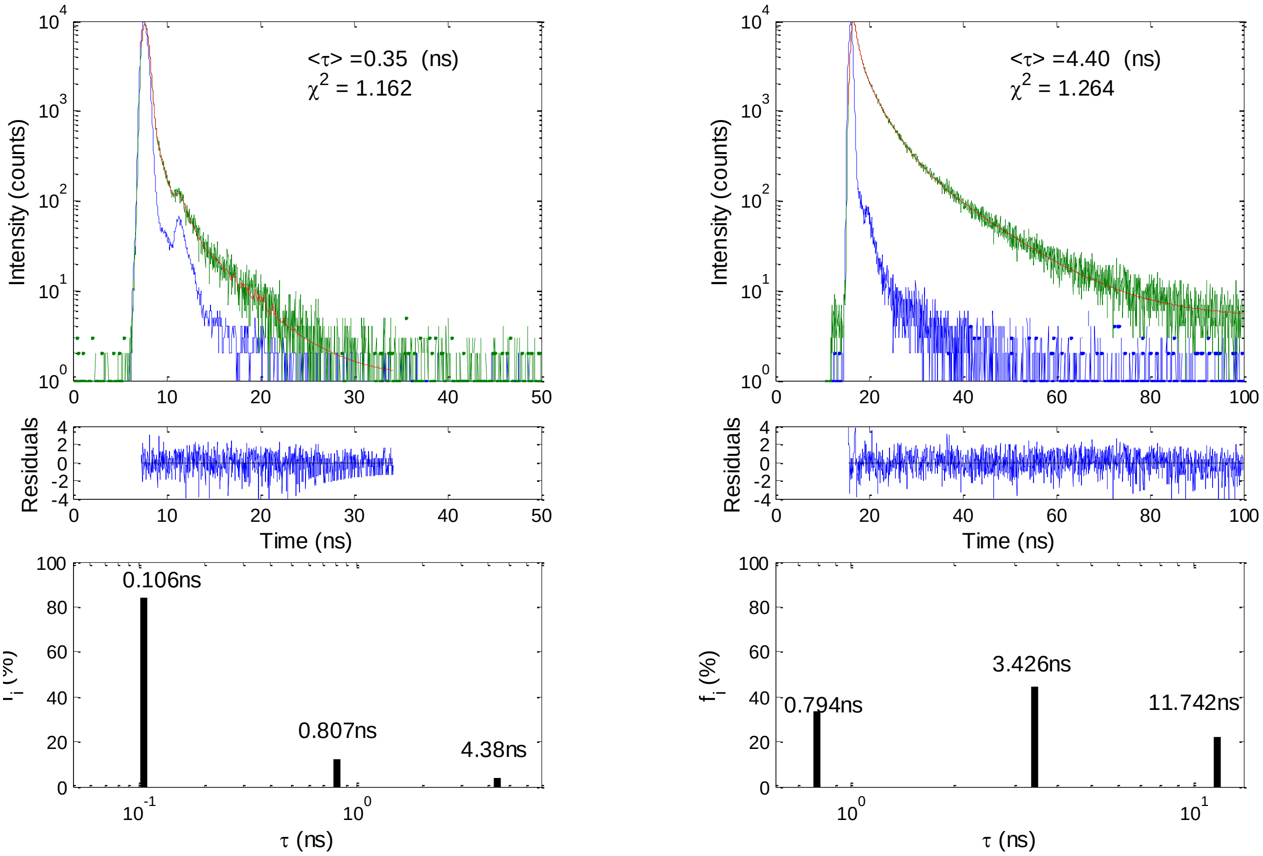

Figure S3j. Fluorescence lifetime measurements of OH-Kyn at 540 nm (left) and its residue in Ac-AA-OH-Kyn-AA-NH_2 at 570 nm (right) upon 442 nm excitation. The data were fitted by 3-exponential models: OH-Kyn (τ_1 = 0.1 ns (84.1%), τ_2 = 0.8 ns (12.1%) and τ_3 = 4.4 ns (3.8%), average lifetime 0.35ns); Ac-AA-OH-Kyn-AA-NH_2 (τ_1 = 0.8 ns (33.4%), τ_2 = 3.4 ns (44.3%) and τ_3 = 11.7 ns (22.3%), average lifetime 4.4ns).

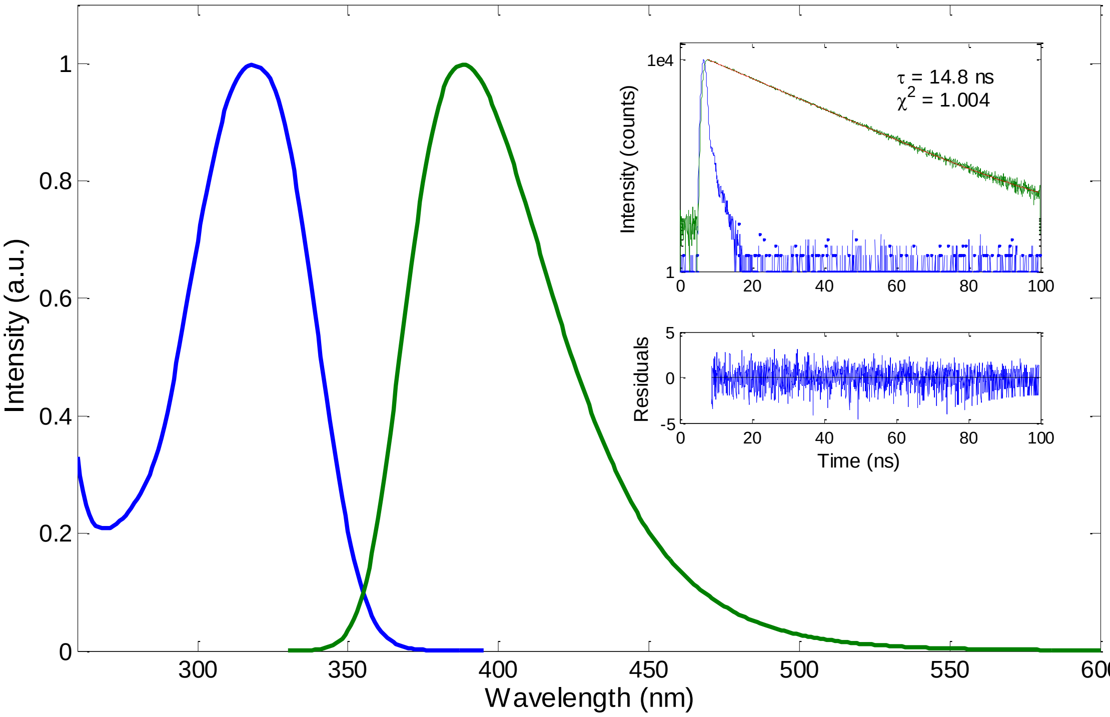

Figure S3k. Fluorescence excitation (blue) and emission (green) spectra and lifetime measurements (inset) of ArgP in PBS. The fluorescence time-response was measured at 390 nm upon 317 nm excitation and fitted by a single-exponential model (τ = 14.9 ns).

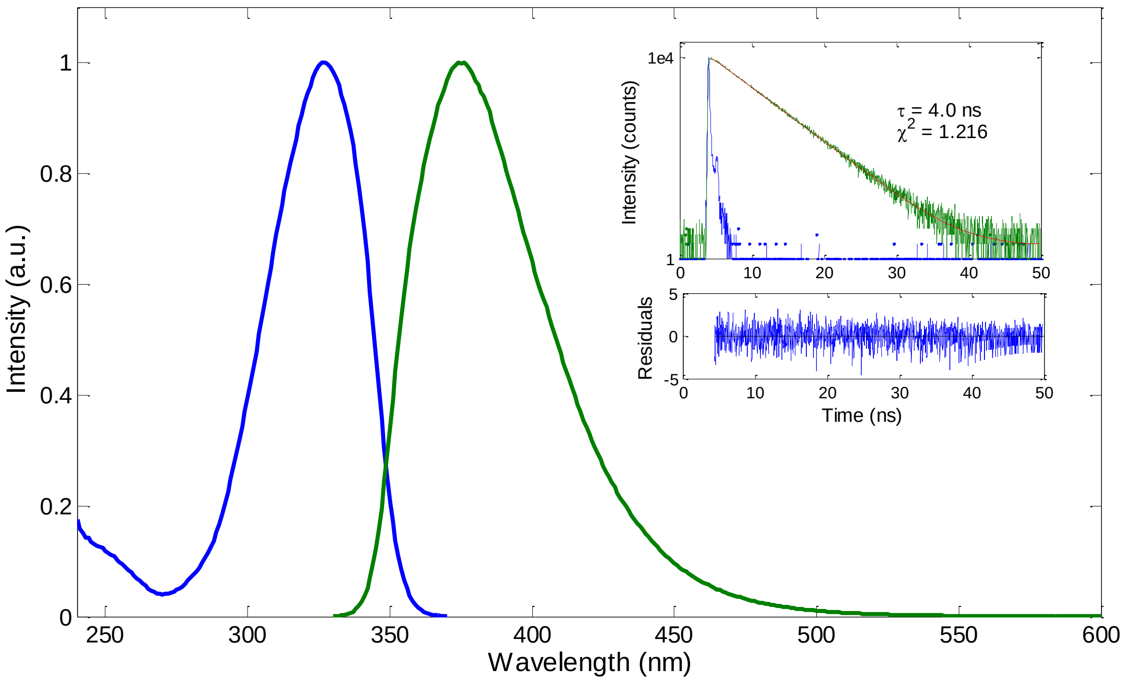

Figure S3l. Fluorescence excitation (blue) and emission (green) spectra and lifetime measurements (inset) of Pentosidine in PBS. The fluorescence time-response was measured at 370 nm upon 315nm excitation and fitted by a single-exponential model (τ = 4.0 ns).

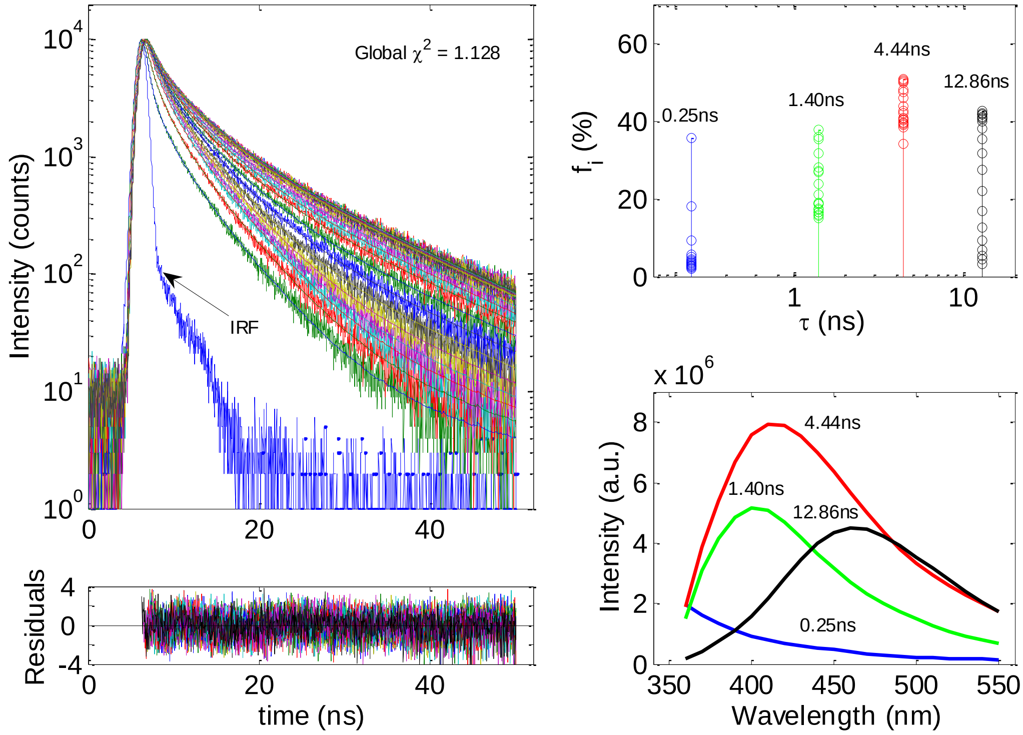

Figure S4. Global analysis of fluorescence time-responses (left panel) measured in the insoluble fraction of a NC++ sample upon 317nm excitation in the 350 – 550nm range. The analysis was carried out by the FAST software (Edinburgh Instruments, UK) by a 4-exponentil model with "linked" lifetime parameters. Graphical representation of the lifetime components (right top panel). Spectra associated with the lifetime components (right bottom panel).

Supplemental Figure S5

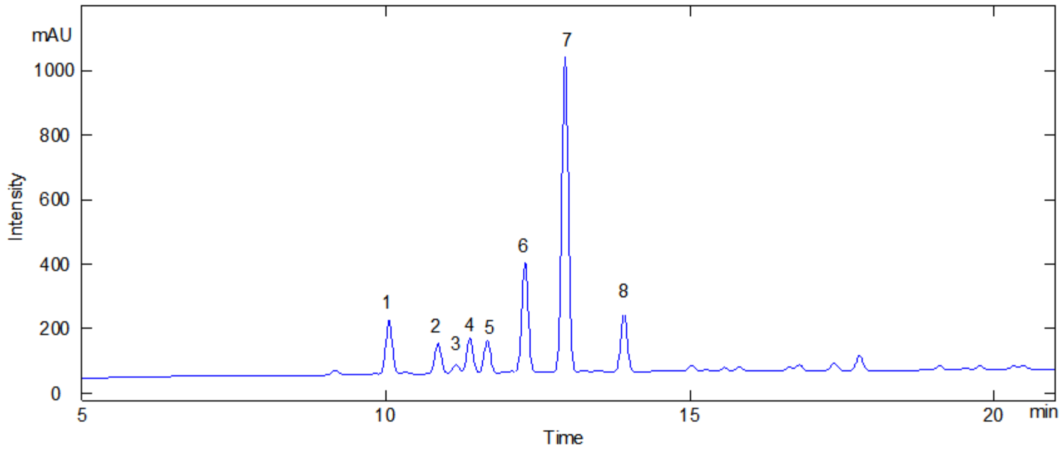

Figure S5. Reversed phase chromatogram of UV-irradiated Ac-AAWAA-NH_2 peptide in 0 - 80% Acetonitrile gradient at flow rate 1ml/min in C18 column.

Supplemental Figure S6

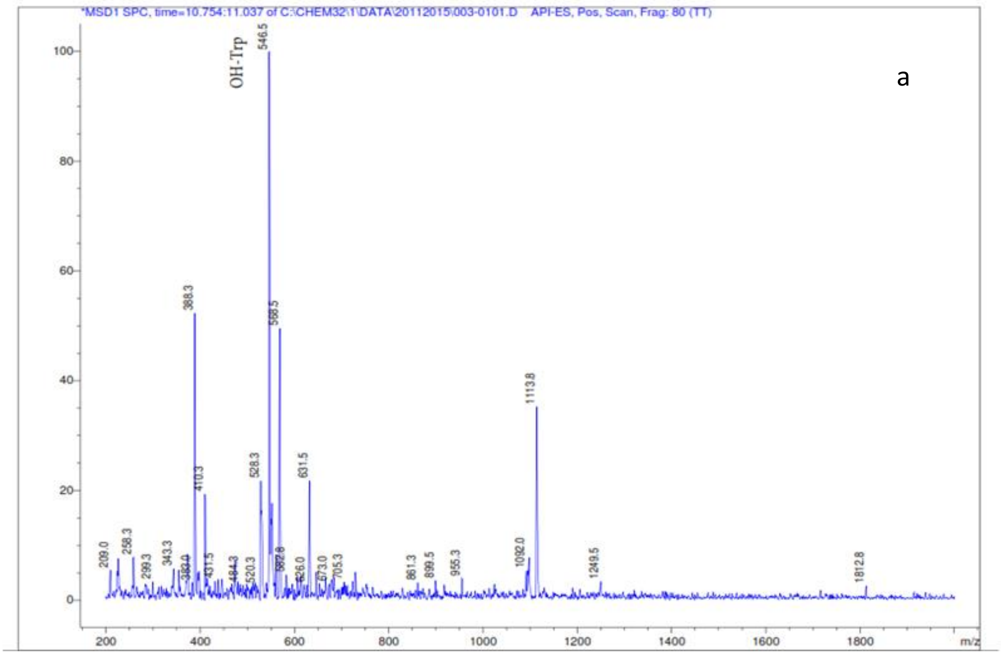

Fraction 2

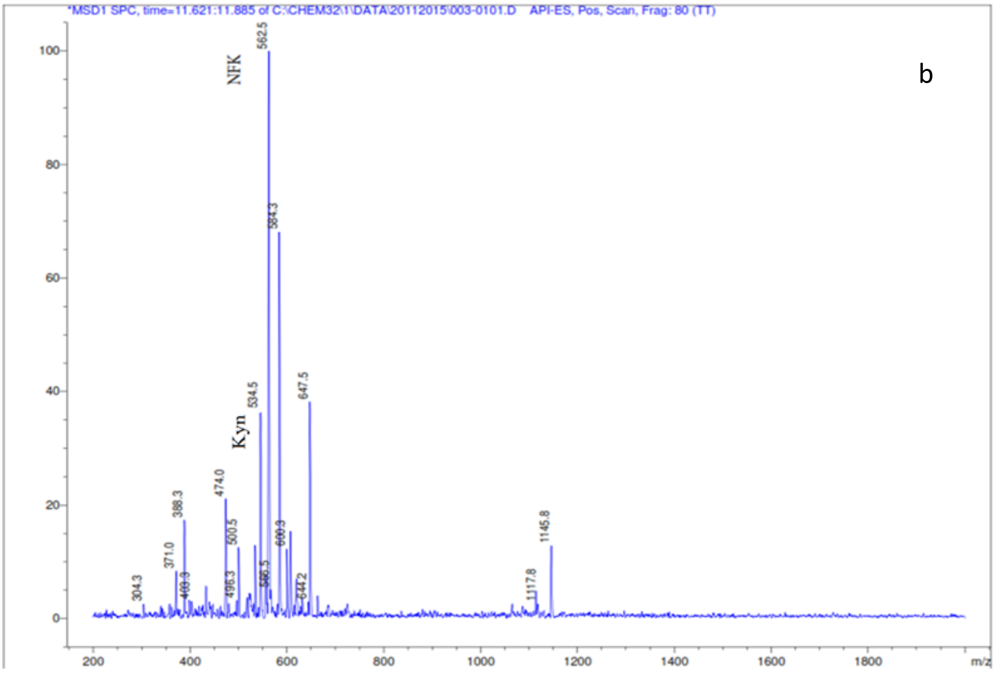
*MSD1 SPC, time=11.621:11.885 of C:\CHEM32\1\DATA\20112015\003-0101.D API-ES, Pos, Scan, Frag: 80 (TT)
b
NFK
Kyn
562.5
584.3
534.5
647.5
474.0
388.3
500.5
600.3
371.0
1145.8
304.3
403.3
498.3
566.5
644.2
1117.8
100
80
60
40
20
0
200
400
600
800
1000
1200
1400
1600
1800
m/z

Fraction 5

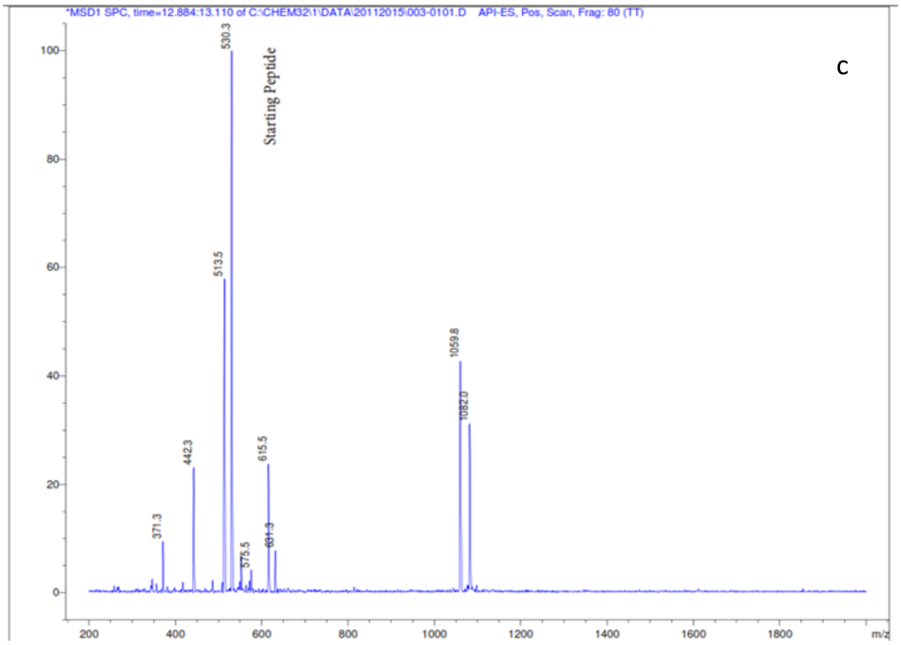
*MSD1 SPC, time=12.884:13.110 of C:\CHEM32\1\DATA\20112015\003-0101.D API-ES, Pos, Scan, Frag: 80 (TT)
c
Starting Peptide
530.3
513.5
1059.8
1082.0
442.3
615.5
371.3
631.3
575.5
m/z

Fraction 7

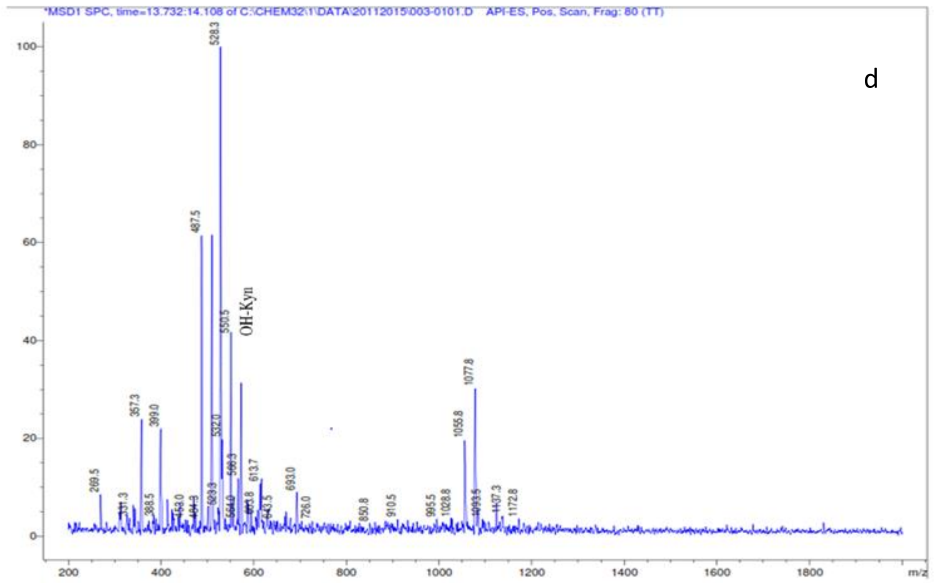

Fraction 8

Figure S6. Mass spectra of the HPLC fractions shown in Fig. S5 containing fluorescent Trp derivatives. The experiments were performed on a Bruker Daltonics mass spectrometer using electrospray ionisation in positive ion mode.

A. Decomposition of the Fr5 fluorescence spectrum

Fluorescence lifetime measurements have been employed to decompose this spectrum into its distinct components. To this end we measured fluorescence time-responses along the spectrum in the 400 – 600 nm range upon 372 nm picosecond excitation. It was found that the emission time-responses on the red slope (Fig. 4D, red) were bi-exponential with major 7.8ns (71.1%) and minor 4.1ns (28.9%) lifetime components (inset, red). Based on spectral position this was attributed to emission of AA-Kyn-AA. The emission response on the blue slope (Fig. S7, left panel, blue) containing 0.8ns (52.5%), 2.8ns (43%) and 7.0ns (4.5%) lifetime components (inset, blue) is likely to be predominantly caused by emission of AA-NFK-AA.

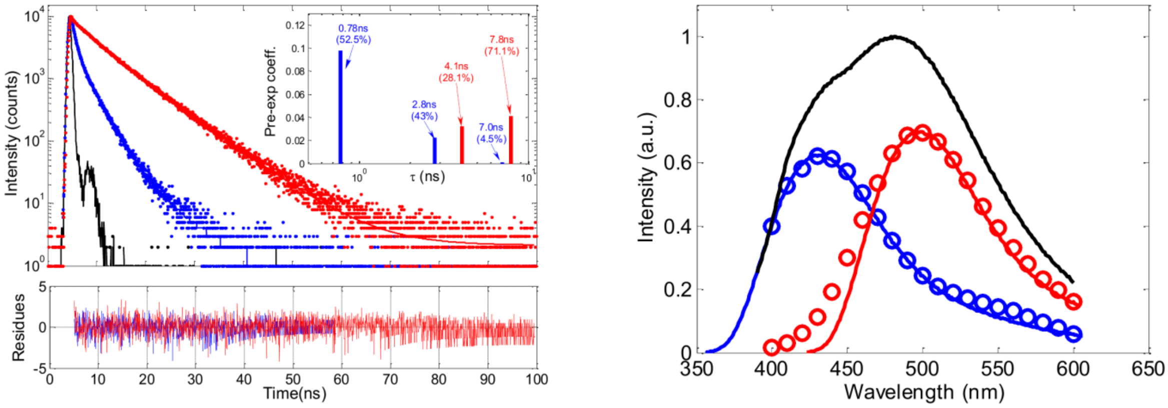

Fig. S7. Fluorescence time-responses of Fr5 measured at 410nm (blue) and 540nm (red) upon 372nm excitation and Instrument Response Function (black) (left panel). Graphical representation of the model parameters for the 410nm (blue) and 540nm (red) time-responses (inset). Decomposition of the emission spectrum of Fr5 measured upon 372nm excitation (black) using spectra associated with τ_1,τ_2 and τ_3 (blue circles) and τ_4 (red circles) lifetime components. The parameters were calculated by evaluating fluorescence time-responses of Fr5 in the 400 - 600nm range by a 4-exponential model with "linked" lifetime parameters. Blue-shifted by 5nm fluorescence spectra of NFK (blue) and fluorescence spectrum of Kyn (red) in PBS (right panel).

To calculate the shapes of the individual spectral bands all these time-responses were evaluated jointly, or "globally", suggesting that the lifetime parameters have the same values for every time-response. In this case, the "global" evaluation ideally required a 5-exponential model in accord with the number of lifetime components.

Evaluation of lifetime data is an ill-posed mathematical problem. The maximal number of lifetime components which the fluorescence lifetime analysis software, the FAST, can handle is four. In addition, it can confidently resolve components with lifetime constants which differ at least 2-fold. In this case the analysis is complicated by the number of components in the exponential model and by the insufficient separation of the minor 4.1 ns component of the red slope emission and the intermediate 2.8 ns component of the blue slope emission and the minor 7.0 ns component of the blue slope emission and the dominant 7.8 ns component of the red slope emission.

Luckily, in this case, the relative contribution of the minor 7.0 ns (3.8%) component of the blue slope was substantially smaller than that of the dominant 7.8 ns component (71.1%) of the red slop therefore enabling the latter to be assigned to the emission of AA-Kyn-AA. These 7.0 ns and 7.8 ns components are given by a single component with a lifetime constant of 7.64 ns in the global analysis by a 4-exponential model (Fig. S8).

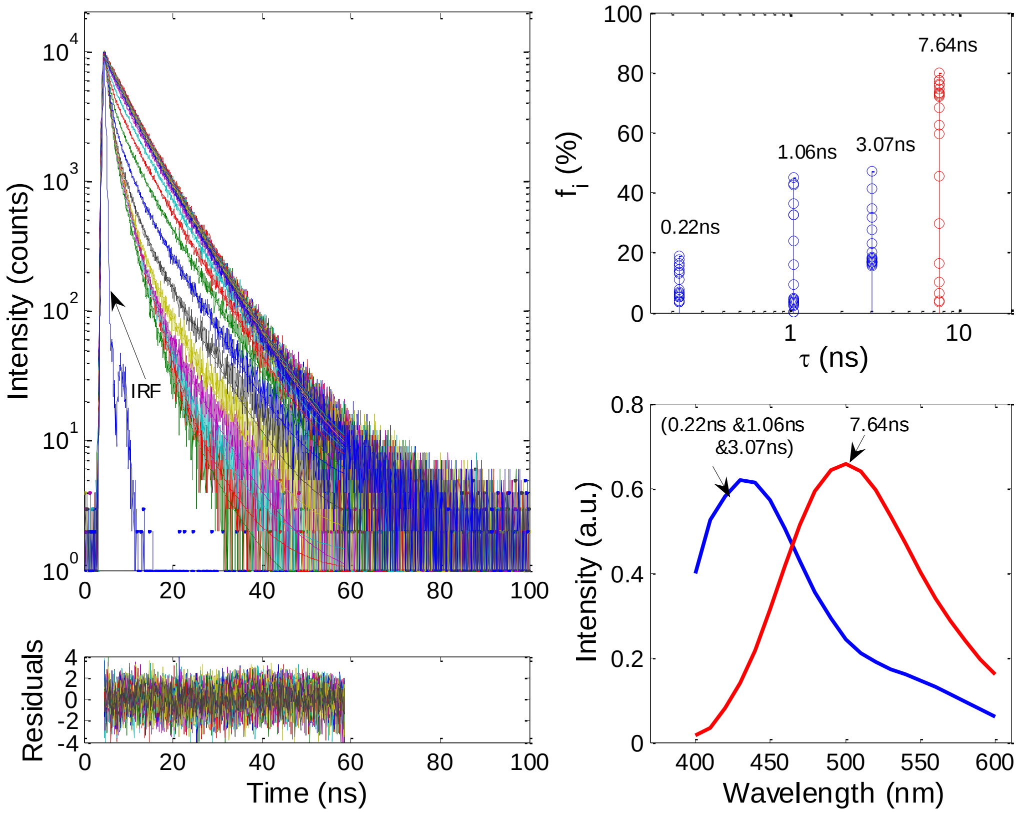

Fig. S8. Global analysis of fluorescence time-responses (left panel) measured in the HPLC Fr5 containing a mixture of Ac-AA-NFK-AA-NH_2 and Ac-AA-Kyn-AA-NH_2 peptides upon 372nm excitation (vertical polarization) in the 350 – 550nm range at the magic angle. The analysis was carried out by the FAST software by 4-exponentil model with "linked" lifetime parameters. Graphical representation of the lifetime components (right top panel). Spectra associated with the first three (0.22ns, 1.06 and 3.07ns) (blue) and the forth (7.64ns) (red) lifetime components (right bottom panel).

Assuming that the red slope emission is homogeneous, which implies that the ratio of fluorescence intensity of these two components (4.1 ns and 7.8 ns) remains constant along the spectrum, we can use the pre-exponential coefficient of the resolved 7.64 ns component to calculate the emission spectrum of the Kyn-derivatised peptide.

The remaining three lifetime components of the global analysis (0.22ns, 1.06ns, and 3.07ns) we can assign with a degree of accuracy to the emission of AA-NFK-AA. Spectra associated with the first three lifetime components (blue circles) and the fourth component (red circles) are shown in Fig. S7 (right panel). The spectrum with a maximum at ~500 nm corresponds to the emission spectrum of Kyn (red solid line). The second decay associated spectrum was 5 nm blue-shifted from that of "free" NFK (blue solid line) and hence was attributed to emission of the NFK-containing peptide. The mismatches on the slopes of the decay associated spectra are due to the above mentioned assumptions in the data evaluation (the "cross-talks" between the 2.8ns and 4.1ns and 7.8 ns and 7.0 ns components and the use of the 4-exponential model).

Table S1. Comparison of average fluorescence lifetime of Trp and its derivatives in “free” and residue form in PBS.

Molecule	Average fluorescence lifetime
NATA	2.9ns
AAWAA	1.7ns
OH-Trp	3.4ns
AA-OH-Trp-AA	4.5ns
NFK	0.16
AA-NFK-AA	2.2ns
Kyn	60ps
AA-Kyn-AA	6.9ns
OH-Kyn	0.35ps
AA-OH-Kyn-AA	4.4ns
